# 500‐Fold Amplification of Small Molecule Circularly Polarised Luminescence through Circularly Polarised FRET

**DOI:** 10.1002/anie.202011745

**Published:** 2020-12-01

**Authors:** Jessica Wade, Jochen R. Brandt, David Reger, Francesco Zinna, Konstantin Y. Amsharov, Norbert Jux, David L. Andrews, Matthew J. Fuchter

**Affiliations:** ^1^ Department of Chemistry and Molecular Sciences Research Hub Imperial College London White City Campus, 82 Wood Lane London W12 0BZ UK; ^2^ Institute for Molecular Science and Engineering and Centre for Processable Electronics Imperial College London South Kensington Campus London SW7 2AZ UK; ^3^ Department Chemie und Pharmazie & Interdisciplinary Center for Molecular Materials (ICMM) Friedrich-Alexander-Universität Erlangen-Nürnberg Nikolaus-Fiebiger-Strasse 10 91058 Erlangen Germany; ^4^ Dipartimento di Chimica e Chimica Industriale Università di Pisa Via Moruzzi 13 56124 Pisa Italy; ^5^ Institute for Organic Chemistry Martin-Luther-Universität Halle-Wittenberg Kurt-Mothes-Straße 2 06120 Halle Germany; ^6^ University of East Anglia Norwich Research Park Norwich NR4 7TJ UK

**Keywords:** chirality, circular dichroism, FRET, helical structures, polymers

## Abstract

Strongly dissymmetric circularly polarised (CP) luminescence from small organic molecules could transform a range of technologies, such as display devices. However, highly dissymmetric emission is usually not possible with small organic molecules, which typically give dissymmetric factors of photoluminescence (*g*_PL_) less than 10^−2^. Here we describe an almost 10^3^‐fold chiroptical amplification of a π‐extended superhelicene when embedded in an achiral conjugated polymer matrix. This combination increases the |*g*_PL_| of the superhelicene from approximately 3×10^−4^ in solution to 0.15 in a blend film in the solid‐state. We propose that the amplification arises not simply through a chiral environment effect, but instead due to electrodynamic coupling between the electric and magnetic transition dipoles of the polymer donor and superhelicene acceptor, and subsequent CP Förster resonance energy transfer. We show that this amplification effect holds across several achiral polymer hosts and thus represents a simple and versatile approach to enhance the g‐factors of small organic molecules.

## Graphical abstract

The amplification of circularly polarised (CP) luminescence of a small chiral molecule embedded within an achiral polymer matrix is demonstrated. This amplification (from |*g*_PL_|=0.0003 to 0.15) is observed in several achiral polymer hosts, due to electrodynamic coupling between the electric and magnetic transition dipoles of the donor (polymer) and acceptor (small molecule), which results in efficient CP Förster resonance energy transfer.

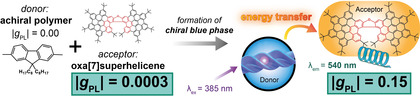

Photonic devices that make use of circularly polarised (CP) light will transform a range of technologies, including data storage, biological sensing, security tags and next‐generation displays.[^1^, ^2^, ^3^, ^4^, ^5^, ^6^] As a result, interest in the identification of materials that can emit CP light (CPL) has surged in recent years.[^7^, ^8^, ^9^] Amongst these materials, small organic chiral molecules offer tuneable electronic properties, simple integration into devices and high fluorescent quantum yields (ϕ_F_).[^10^, ^11^] The *intrinsic* absorption and emission of CP light by molecular systems (so‐called natural optical activity) can be evaluated through the rotational strength, *R*, which is the scalar product of the electric (***μ***) and magnetic (***m***) transition dipole moments [Eq. 1].^[7]^ Here, *θ* describes the angle between ***μ*** and ***m***.

(1) $$R=\left|\mu \right|\cdot \left|m\right|\cos\theta$$

In general, the emission intensities are proportional to the dipole strength, *D*, which is defined as follows for each transition between the emissive state (*j*) and the ground state (*i*) [Eq. 2]:^[12]^

(2) $$D={\left|\langle \Psi_{j}|\mu \pm \left(\frac{i}{c}\right)m|\Psi_{i}\rangle \right|}^{2}$$

where *c* is the speed of light and the ± sign relates to left‐/right‐handed CPL. The dissymmetry of absorption can be described by *g*_abs_ [Eq. 3] and for isolated small organic molecules it has been shown that the photoluminescence (PL) dissymmetry factor (*g*_PL_) is often linearly proportional to, and smaller than, the absorption dissymmetry (*g*_abs_).^[13]^

(3) $$g_{abs}=4\times \frac{R}{D}=\frac{4}{c}\frac{\left|m\right|\left|\mu \right|}{{\mu^{2}+\left(m/c\right)}^{2}}\cos\theta \approx \frac{4}{c}\frac{\left|m\right|}{\left|\mu \right|}\cos\theta$$

For many small organic molecules, a high ϕ_F_ coincides with high *D* [Eq. (2)], but since ***μ*** is considerably larger than ***m***, this results in a very small |*g*_PL_| [Eq. (3)] (10^−4^ to 10^−2^).[^14^, ^15^, ^16^, ^17^] There has therefore been significant interest in identifying ways to escape this seemingly mutually exclusive relationship and realise molecules that are highly emissive *and* emit highly dissymmetric CPL. Recently, amplification of the *g*_PL_ of small chiral molecules by one order of magnitude (from 10^−4^ to 10^−3^) has been achieved through triplet–triplet annihilation‐based upconversion‐induced fluorescence.[^18^, ^19^] In such systems, the excitation of donors that incorporate heavy atoms permits intersystem crossing to triplet states, and their chirality results in the generation of spin‐polarised triplet excitons which ultimately enhance the *g*_PL_ of chiral acceptors.^[18]^ For small molecules which self‐assemble into anisotropic aggregates, Zinna, Di Bari and co‐workers have shown it is possible to achieve high |*g*_PL_| (10^−2^) through a coupling of linear fluorescence anisotropy and linear birefringence.^[20]^ Alternatively, efforts have been made to induce chiroptical effects into otherwise achiral molecules, primarily through the incorporation of carefully selected achiral molecules into chiral media. For example, Zhu, Liu et al. demonstrated the induction of CPL (|*g*_PL_| ≈10^−2^) of achiral small molecule luminophores when embedded within the chiral voids of a cubic, self‐assembled cyclodextrin metal organic framework.^[21]^

Beyond small molecules, conjugated polymer systems can combine high ϕ_F_ with strong *g*_PL_, most likely due to a combination of long‐range chiral order (e.g. formation of a chiral supramolecular assembly or, in the presence of alignment layers, cholesteric liquid crystalline phases) and natural optical activity (e.g. coupling of excited states on adjacent polymer chains).[^5^, ^6^, ^22^, ^23^, ^24^] Whilst there are several strategies to introduce chirality into conjugated polymer systems, our group and others have shown that a simple and versatile approach is to combine an achiral polymer (ACP) with a chiral small molecule additive.^[9]^ In such blend systems, high *g*_abs_ and *g*_PL_ are observed for emission/absorption from the polymer chromophore, with the chiral small molecule additive (in our case aza[6]helicene, hereafter aza[6]H) only serving as a structural template to guide the system into a left‐ or right‐handed form. We have previously identified that the origins of strong chiroptical phenomena in non‐aligned ACP—chiral additive (ACPCA) systems—lie in natural optical activity.^[25]^ An alternative opportunity would be to create an ACPCA system where the chiral small molecule additive was responsible for the fluorescence, within a polymer matrix.

For over ten years, Förster resonance energy transfer (FRET) has been demonstrated as a means to generate luminescence from an acceptor species embedded within a donor matrix for high performance OLEDs.[^26^, ^27^, ^28^, ^29^] The FRET process occurs due to the coupling of donor and acceptor electric transition dipoles (often represented as V) and involves the non‐radiative transfer of energy from the excited state of the donor to the acceptor in its ground state. Another, parallel (but often weaker) form of coupling involves a transition electric dipole at one component and a magnetic transition dipole at the other, which forms a coupling represented by U.^[30]^ The entire sequence of photoexcitation, energy transfer, and fluorescence involves at least four transition dipoles, two at the donor and two at the acceptor.^[30]^ When considering the coupled dipoles, we follow the work of Andrews et al. and introduce the terms E1 and M1, which refer to the electric (***μ***) and magnetic transition dipole moments (***m***), respectively, and their involvement in coupling.^[31]^ Couplings involving at least one magnetic dipole e.g. (M1E1^3^)U typically have an enhanced CP emission compared to the pure electric dipole coupling, and do not have the same distance‐dependence as V, such that chiral energy transfer does not only occur between nearest neighbours.[^31^, ^32^] The FRET process has been previously shown to generate reasonably weak CPL (|*g*_PL_| ≈3×10^−3^) from achiral small molecule acceptors when incorporated into chiral supramolecular structures such as nanohelices.[^33^, ^34^, ^35^, ^36^]

Here, we describe the highly dissymmetric photoluminescence (|*g*_PL_| >0.1) of an enantiopure chiral π‐conjugated small molecule acceptor (oxa[7]superhelicene, hereafter oxa[7]H) embedded within a range of ACP donors (PFO, F8BT, F8PFB, Figure 1 and Supporting Information, SI). The oxa[7]H (10 wt %) induces a chiral phase within the ACPs—analogous to the phase formed in polymer/aza[6]H blends, which we have previously characterised.[^6^, ^25^, ^37^] The energy levels and luminescence quantum yields of the ACPs are such that efficient FRET can occur to the oxa[7]H. By exciting the donor–acceptor system in the donor absorption band, we observe highly dissymmetric CP emission from the oxa[7]H (|*g*_PL_|>0.1) with a ϕ_F_ of over 50 %. This represents an amplification of the oxa[7]H |*g*_PL_| of almost three orders of magnitude over the values in solution. We propose that this *g*_PL_ amplification originates not primarily by chiral perturbation through the host, but instead from (M1E1^3^)U coupling between the ACP and oxa[7]H, such that the emission of the donor results in circularly polarised FRET to the oxa[7]H. These findings offer a simple and straightforward approach to massively enhance the intrinsic *g*_PL_ of small molecule CP emitters that is compatible with a range of photonic applications.

**Figure 1:**
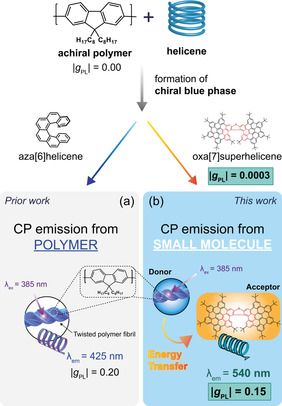
A cartoon to indicate the similarities and differences between our a) prior and b) current work. We previously demonstrated that enantiopure aza[6]helicene can be used to induce a chiroptical response in achiral polymer systems, resulting in CP emission from the polymer. Here, CP FRET amplifies the otherwise weak intrinsic response of the small molecule (*g*_PL_: 0.0003) and result in strong CP emission from the helicene (*g*_PL_: 0.15).

First, we consider the photophysical properties of solutions (Figure S1, S2) and thin films (Figure S3, S4) of the neat, enantiopure oxa[7]H (LUMO: −2.0 eV, HOMO: −4.8 eV calculated using Density Functional Theory with the B3LYP functional at the 6‐31 G(d,p) level of theory). Comparable with the previously reported solution studies, the extended π‐conjugation results in characteristic absorption bands (λ_abs_: 482 nm, 520 nm), which are slightly red‐shifted in thin films compared to solution (λ_abs_: 480, 515 nm) (Figure 2, S1 and S3).[^38^, ^39^] Circular dichroism (CD) and the associated *g*_abs_ spectra reveal several distinct peaks (λ: 233, 355, 509, 530 nm, Figure S1, S3), which are equal‐and‐opposite for the two enantiomers (|*g*_abs_|=2×10^−3^ at 509 nm and ≈10^−4^ at 530 nm). The enantiomers were assigned as [*P*] and [*M*] from the sign of the low energy CD band (Figure S1). The photoluminescence of neat oxa[7]H thin films (Figure S4) is weak and broad, but the vibrational fine structure can still be resolved (λ_em_: 540, 620, and 675 nm). We attribute this broadening and quenching to aggregation, which is caused by the strong π–π stacking tendencies of neat oxa[7]H.^[40]^ CP photoluminescence (CP PL) measurements and the associated *g*_PL_ spectra were recorded for the oxa[7]H enantiomers in solution (Figure S1, S2), revealing clear mirror image bands centred around λ_em_: 540 nm. In order to achieve a more reliable quantification, *g*_PL_ was estimated from the ratio of the integral of CP PL and total PL collected in the 490 to 670 nm range (|g_PL_|=3 ×10^−4^). This value is comparable to the *g*_abs_ of the most red‐shifted Cotton effect at 530 nm (Figure S1) in terms of sign and magnitude; an indication that geometry of the ground and emitting excited state are similar.^[13]^ Equivalent CP PL measurements of as‐cast thin films of neat oxa[7]H were attempted, but relatively strong linear contributions prevented a reliable quantification of any CPL signals with *g*_PL_<10^−3^, making *g*_PL_ values comparable to those measured in solutions (10^−4^) not detectable. The linearly polarised components of the PL signal increased after thermal annealing, likely due to the crystallisation of oxa[7]H, demonstrating a linear dissymmetry [(I_∥_−I_⊥_)/(I_∥_+I_⊥_)] ≈7.5×10^−3^.

**Figure 2:**
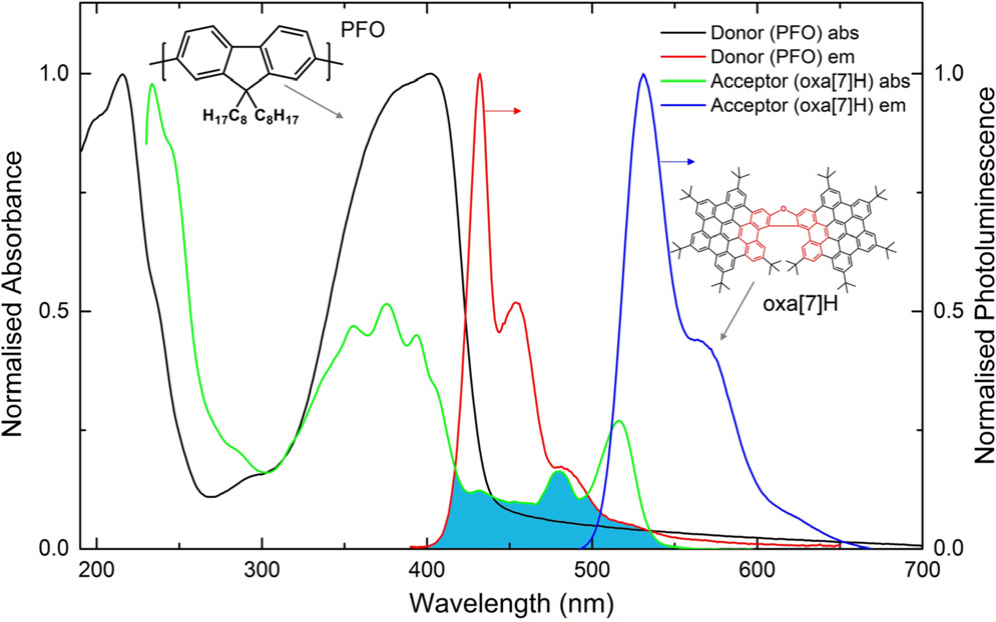
Normalised absorption and photoluminescence (λ_ex_: 385 nm) spectra of thin films of the donor (PFO, thickness, *t*=140 nm) and acceptor (oxa[7]H, *t*=90 nm) systems used in this work. The overlap between the donor emission and acceptor absorption is highlighted in turquoise.

Informed by our previous experience with ACPCA blends, we combined 10 wt % of enantiopure oxa[7]H with PFO (LUMO: −2.1 eV, HOMO: −5.8 eV) (for chemical structures and energy level diagrams see Figure S5). As‐cast PFO:oxa[7]H thin films display absorption spectra characteristic of the neat polymers, with PFO displaying two clear absorption bands (λ_abs_: 217 nm, 390 nm) in this spectral range (Figure 3, Figure S5). In contrast to emission of the neat PFO (Figure 2), the PL of the PFO:oxa[7]H blends (Figure 4) is reminiscent of the solutions of neat oxa[7]H, irrespective of whether the blend is excited at the ACP or oxa[7]H absorption maximum. For example, the PL of the PFO:oxa[7]H blends is dominated by oxa[7]H emission at λ_em_: 530, 570, and 620 nm, with only a weak (<8 % of the total PL) high energy emission of the characteristically blue PFO (λ_em_: 417, 436, and 464 nm, Figure S7). Regarding the chiroptical response of the blend films, the as‐cast PFO:oxa[7]H blend films display a weak chiroptical response (CD ≈5 mdeg) similar to the thin films of neat oxa[7]H (Figure 3), which is equal‐and‐opposite for the enantiomers. CP PL measurements of the as‐cast PFO:oxa[7]H blends reveal negligible dissymmetry (*g*_PL_ <10^−2^).

As for our previous ACPCA systems, thermal annealing of the ACP:oxa[7]H thin films leads to a strong increase in the strength of the chiroptical response. In situ temperature‐dependent CD spectroscopy (Figure S8, S9) showed that the PFO:oxa[7]H thin films are CD silent below 140 °C, above which there is a dramatic increase in the chiroptical response. This large induced CD, which is equal‐and‐opposite for the blends containing [*P*] and [*M*]‐oxa[7]H enantiomers, reaches a maximum at high temperatures (*T*_CD Max_: 160 °C), and is retained upon cooling to room temperature. With this insight, we followed a similar protocol to our previous studies using aza[6]H (annealing at *T*_CD Max_ for 10 minutes in the glovebox and quenching to room temperature), and investigated the photophysical properties of the annealed PFO:oxa[7]H thin films (Figure S10).[^6^, ^25^]

The absorption band of the annealed PFO:oxa[7]H blends (Figure 3) is considerably broader than their as‐cast counterparts (Δ FWHM: +23 nm), with a low‐energy shoulder appearing at 398 nm. The CD spectra contain an extremely strong (18,000 mdeg, *g*_abs_ >1.5 at 410 nm) bisignate couplet centred close to the absorption maximum (375 nm), which is positive for the *P* oxa[7]H enantiomer and negative for *M* (Figure 3, Figure S11). This CD response is one of the highest ever reported for polymer thin films, and six times greater than our previous studies of PFO:aza[6]H (CD ≈3,100 mdeg).^[25]^ The CD spectrum also shows signals similar to the oxa[7]H solution spectrum (λ_abs_: 482 nm, 518 nm), with equal‐and‐opposite CD bands corresponding to the low energy absorption bands of oxa[7]H (CD ≈500 mdeg, 3 % of the integrated CD signal).

**Figure 3:**
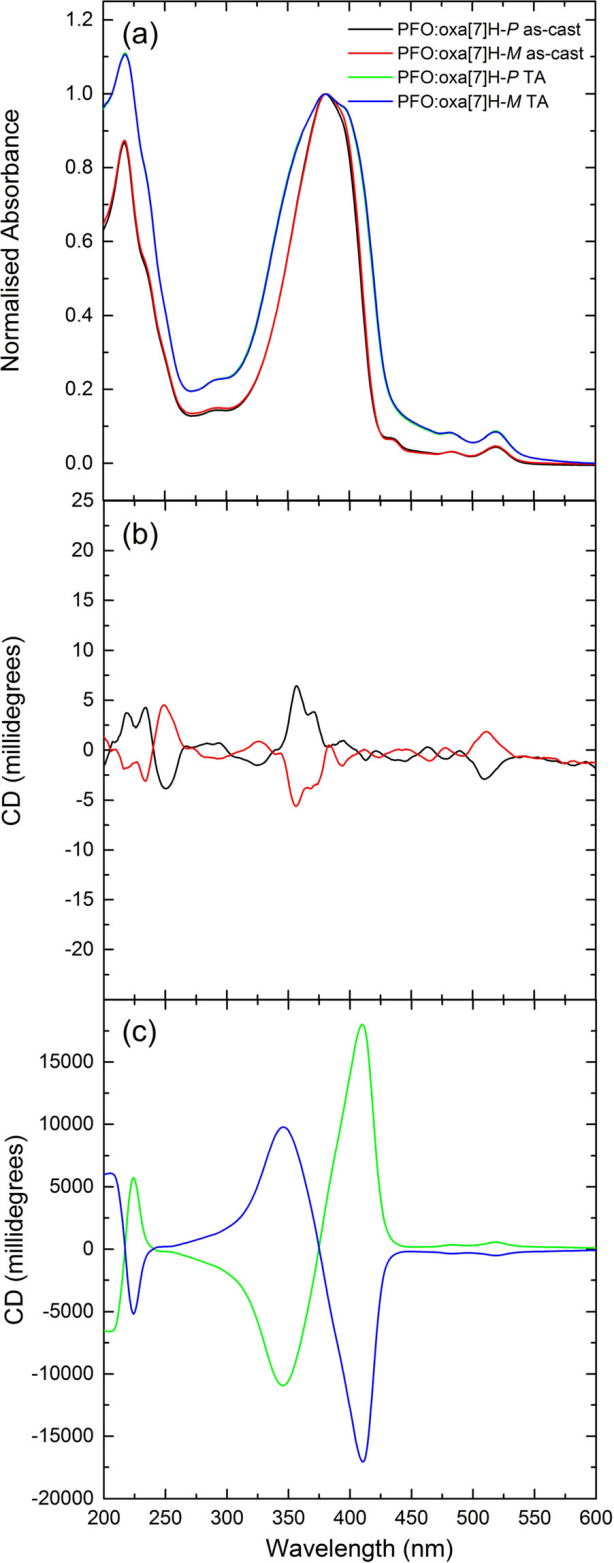
a) Absorbance and b,c) circular dichroism spectra of as‐cast (black, red) and annealed (160 °C, 10 minutes in an N_2_ glovebox) (green, blue) PFO:oxa[7]H thin films (*t*=135 nm).

To ensure that linear artefacts did not contribute to the measured chiroptical response, we performed Mueller Matrix Spectroscopic Ellipsometry (MMSE), allowing us to decouple the linear and circular dichroism and birefringence in both transmission and reflection (Figure S12, S13). The transmission MMSE spectra of annealed PFO:oxa[7]H films reveal strong circular terms (MM_14_, MM_23_, MM_32_), which are equal‐and‐opposite for the blends containing oxa[7]H enantiomers, and absent from the reflection spectra. The linear terms in the reflection spectra (MM_12_, MM_21_, MM_33_) are the result of uniaxial anisotropy, where the optical axis of the polymer aligns perpendicular to the surface, and can be described by a diagonal dielectric tensor.^[25]^ We have previously shown that such terms do not contribute to the chiroptical response.^[25]^

For the annealed PFO:oxa[7]H systems (Figure 4), the PL (λ_ex_: 385 nm) retains the emission maxima and vibrational fine‐structure of the as‐cast films, but with very weak evidence of PFO emission (λ_em_: 425, 440 and 451 nm, 1 % of the integrated PL signal, Figure S14) at high energy. Instead, as for the as‐cast films, the PL is dominated by emission from the oxa[7]H. CP PL measurements of the annealed PFO:oxa[7]H blends reveal that this emission from oxa[7]H is unexpectedly highly dissymmetric; with a clear bisignate luminescence dissymmetry (|*g*_PL_| PFO:oxa[7]H blends ≈0.15, Figure 4), centred at λ_em_: 540 nm. The bisignate signal in the CPL spectra (Figure 4 (d), centred at λ_em_: 540 nm) may result from circular‐selective self‐absorption due to the CD response of oxa[7]H at these wavelengths (Figure 4, Figure S14 and accompanying discussion). The ϕ_F_ of the PFO:oxa[7]H systems, calculated using the method developed by De Mello et al.,^[41]^ increases slightly after annealing (49.3 to 53.2 %, Table S1). To investigate the influence of donor versus acceptor excitation, we excited the PFO:oxa[7]H blend in a region where only the oxa[7]H absorbs (λ_ex_: 515 nm) (Figures S15–S17), and a greatly reduced CPL signal (|*g*_PL_| ≈10^−2^) was observed.

**Figure 4:**
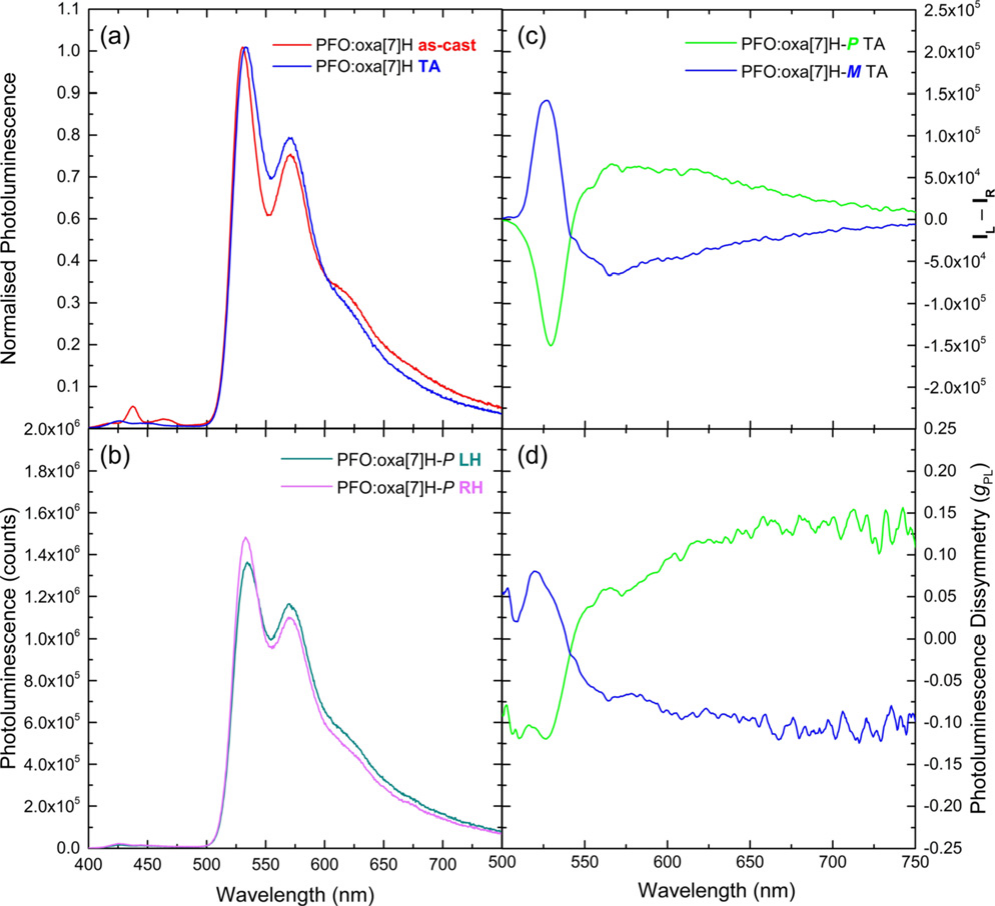
a) As‐cast (red) and annealed (160 °C, 10 minutes in an N_2_ glovebox) (blue) PL spectra (λ_ex_: 385 nm) for PFO:oxa[7]H blends (*t*=135 nm), b) LH and RH PL spectra for PFO:oxa[7]H and the c) CP PL and d) associated *g*_PL_ spectra of annealed PFO:oxa[7]H [*P*] (green) and [*M*] (blue) thin films.

We next compared the surface topography of the PFO and PFO:oxa[7]H thin films using Atomic Force Microscopy (AFM, Figure S18), and find that not only are the blend systems remarkably smooth (root‐mean‐square roughness=0.2 nm), but they resemble the annealed, non‐aligned ACPCA films we have studied previously.^[25]^ The fluorescence lifetime of PFO is dramatically reduced in the PFO:oxa[7]H blends compared to their neat counterparts (Figure S19), which indicates that the FRET process is highly efficient.

To demonstrate the general nature of this approach, we repeated these measurements with other ACP donors, including F8BT and F8PFB (Figure 5, Figures S21–S33). The results, discussed in more detail in the SI, show that in both cases a similar chiral phase is formed in the ACPs upon annealing (CD ≈6,000 mdeg, Figure 5 a–d, Figure S23, S29). As with the PFO:oxa[7]H blends, the resolved vibronic structure and emission energies of the ACP:oxa[7]H blend thin films indicate that the PL is dominated by the oxa[7]H (Figure 5). For both annealed ACP:oxa[7]H blends, the oxa[7]H PL is strongly dissymmetric, |*g*_PL_| F8BT:oxa[7]H ≈0.15, F8PFB:oxa[7]H ≈0.07. The fact that the PL is dominated by emission from oxa[7]H, coupled with the similar *g*‐factors for PFO and F8BT (Figure 4 and 5), hint that in both blend films the same FRET mechanism is at play.

**Figure 5:**
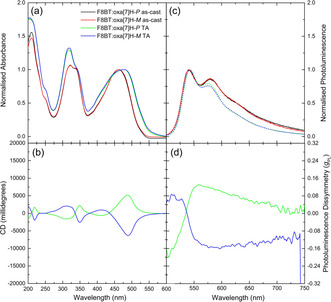
a) Absorbance, b) circular dichroism, c) photoluminescence and d) photoluminescence dissymmetry of neat and annealed (*T*=140 °C, 10 minutes) F8BT:oxa[7]H (*t*=131 nm, λ_ex_: 475 nm). Further systems are considered in the Supporting Information.

The in situ and steady‐state CD measurements (Figure 3, Figure 5, Figures S8–S10, S21–S23 and S29) indicate that a thermodynamically stable chiral phase is formed in the ACP:oxa[7]H thin films upon annealing, with a handedness corresponding to the handedness of the oxa[7]H. The induced chiroptical signal in PFO was approx. 6‐fold larger than that achieved with aza[6]H, despite using a five‐fold lower molarity solution of the oxa[7]H.[^6^, ^25^] Taken together, the in situ CD, CD, MMSE, AFM and UV/Vis data indicate that the annealed ACP:oxa[7]H blend systems adopt a similar weakly ordered double twist cylinder blue phase to the non‐aligned ACPCA systems previously investigated by our group.^[25]^ The increased thermodynamic stability and elevated *T*_CD Max_ of the PFO:oxa[7]H blend film compared to our previously reported aza[6]H‐based blends is likely due to both a higher racemisation barrier and a reduced mobility of the oxa[7]H, caused by its larger size and the presence of bulky ^*t*^Bu groups.^[42]^

The dissymmetric PL (|*g*_PL_| ≈0.15, Figure 4, 5) of the annealed ACP:oxa[7]H blends originates from oxa[7]H (Figure 4, Figure S24, Figure S28) and is almost three orders of magnitude higher than recorded for the neat oxa[7]H solutions or thin films (Figure S2, |*g*_PL_| ≈10^−4^). The much lower dissymmetric emission when oxa[7]H is excited directly within the blends (λ_ex_: 515 nm, Figure S15—S17), suggests that the CPL amplification is not primarily caused by a chiral perturbation through the host matrix, but instead through efficient CP FRET between the ACP donor and acceptor. The high absorption coefficients and ϕ_F_s of the ACPs likely enhance this FRET process.^[43]^ The E1–M1 coupling that gives rise to CP‐FRET is most effective as a short‐range interaction, though its power dependence on distance is less acute than E1–E1 coupling (1/*R* ^2^, compared to 1/*R* ^3^ for E1–E1), and it is enhanced when donor and acceptor units are orientationally correlated.^[31]^ The absence of CP PL in the as‐cast films may therefore be due to a) non‐optimised distribution of the oxa[7]H in the ACP, which results in poor circularly polarised FRET, b) the double twist cylinder blue phase not having formed or c) weak CD, which results in weak CP electrodynamic coupling if the ground and excited states have similar geometries. In the annealed ACP:oxa[7]H blends, chiral energy transfer from the ACP (donor) results in the generation of strong CP PL from the oxa[7]H (ϕ_F_ ≈53 %, Table S1). Given the multiple processes involved with FRET, it is perhaps remarkable that the ϕ_F_ remains so high (ϕ_F_ solution ≈80 %) and that energy is not significantly lost to non‐radiative pathways.^[38]^

In conclusion, we present a system where blending a fluorescent chiral small molecule acceptor (oxa[7]H) with ACP donors results in strong CPL from the acceptor. We believe that the mechanism by which this occurs is as follows; upon annealing, the oxa[7]H induces a chiral phase in the ACP, which results in highly dissymmetric absorption (|*g*_abs_| ≈1.5). FRET from the excited state of the ACP to the ground state of the oxa[7]H leads to photoemission from oxa[7]H, with only negligible contributions from the polymer. In the annealed ACP:oxa[7]H blends, this photoluminescence from oxa[7]H is highly dissymmetric (|*g*_PL_| ≈0.15, ϕ_F_ >50 %), far exceeding the |*g*_PL_| of the isolated oxa[7]H molecules (≈10^−4^). This enhancement is not directly caused by the chiral environment of the host. Instead, we attribute it to E1–M1 coupling between the donor and acceptor species, which, after the formation of the double twist cylinder blue phase in the polymer donor, results in efficient chiral energy transfer to the oxa[7]H acceptor. The work described herein may offer a universal strategy for amplifying chiroptical responses of small emissive molecules, reducing the need to optimise chiral emitters for both high QY and high solution dissymmetry. Instead, chiral small molecule luminophores could be selected based on their non‐chiral photophysical properties alone, with even small intrinsic dissymmetries amplified to *g*‐factors >0.1 by the polymer host.

## Conflict of interest

M. Fuchter is an inventor on a patent concerning chiral blend materials (WO2014016611).

## Supporting information

As a service to our authors and readers, this journal provides supporting information supplied by the authors. Such materials are peer reviewed and may be re‐organized for online delivery, but are not copy‐edited or typeset. Technical support issues arising from supporting information (other than missing files) should be addressed to the authors.

Supplementary
