# Supplementary material for: 500‐Fold Amplification of Small Molecule Circularly Polarised Luminescence through Circularly Polarised FRET
Source: Angew Chem Int Ed Engl. 2020 Dec 1;60(1):222–7. doi: 10.1002/anie.202011745 (PMC7839560; doi:10.1002/anie.202011745)
Supplement: Supplementary file 1 — Supplementary [file ANIE-60-222-s001.pdf]

Angewandte Chemie
Eine Zeitschrift der Gesellschaft Deutscher Chemiker

Supporting Information

500-Fold Amplification of Small Molecule Circularly Polarised Luminescence through Circularly Polarised FRET

*Jessica Wade, Jochen R. Brandt, David Reger, Francesco Zinna, Konstantin Y. Amsharov, Norbert Jux, David L. Andrews, and Matthew J. Fuchter**

anie_202011745_sm_miscellaneous_information.pdf

Contents

Methods 4

Neat oxa[7]H 6

Solution UV-Vis/ PL measurements 6

Thin film UV-Vis/ PL measurements..8

Materials used in this work 10

PFO:oxa[7]H Blends..11

Photoluminescence..11

In situ Circular Dichroism 12

Circular Dichroism..13

Mueller Matrix Spectroscopic Ellipsometry 15

PLQY measurements...16

CP PL measurements 17

Atomic Force Microscopy...20

Excited state dynamics 21

F8BT:oxa[7]H 22

In situ Circular Dichroism 23

Dissymmetric absorption/photoluminescence 24

Discussion of F8BT results...26

F8:PFB:oxa[7]H..27

UVVis/PL 28

PLQY measurements...29

Dissymmetric absorption/photoluminescence 30

Mueller Matrix Spectroscopic Ellipsometry Measurements..32

Discussion of F8PFB results 33

References 34

Methods

Materials:

The conjugated polymers used in this study include poly(9,9-di-n-octylfluorenyl-2,7-diyl), **PFO** (M_P = 59 K), poly (9, 9-dioctylfluorene-alt-benzothiadiazol), **F8BT** (M_N = 9 K), poly(9,9-dioctylfluorene-*alt*-bis-*N*,*N′*-(4-butylphenyl)-bis-*N*,*N′*-phenyl-1,4-phenylenediamine with a volume fraction of 95% F8 and 5% PFB units and **F8PFB** (M_P = 114 K). They were provided by Cambridge Display Technology. Oxa[7]H was prepared as previously reported[1] and separated using preparative chiral HPLC.

Solution photophysical characterisation:

CPL measurements were carried out with a home-made spectrofluoropolarimeter.[2] The concentration of the samples was around $3 \cdot 10^{-6}$ M in CH_2Cl_2. A 365 nm LED was used as the excitation source, employing a 90° geometry and the excitation light was linearly polarized parallel to the detector. Acquisition parameters were as follows: bandwidth ~5 nm, scan-speed 1 nm/sec, integration time 4 sec, photomultiplier voltage 500 V, accumulations 6.

Solid state CPL measurements of neat oxa[7]H were attempted by using a 0° geometry between excitation (unpolarized λ_{ex} = 365 nm) and light collection. ECD spectra were measured with a Jasco J-715 spectropolarimeter in 1 cm cell in $1 \cdot 10^{-5}$ M CH_2Cl_2 solutions ($1 \cdot 10^{-4}$ M to highlight the signals in the lower energy region).

Solution Preparation and thin film deposition: PFO, F8PFB and F8BT were dissolved with a fixed ratio of oxa[7]H (10 wt%) in toluene to form 30 mg/mL solution. Quartz substrates (UQG) were rinsed in an ultrasonic bath with acetone, isopropyl alcohol (Sigma Aldrich) and Hellmanex III (Hellma GmbH) and deionized water, before being treated in an ozone plasma asher (100 W, 3 minutes) and dried with a nitrogen gun. Thin films were fabricated *via* spin-coating (spin-speed 1600 rpm). Samples were annealed for 10 min under nitrogen atmosphere (glovebox, H_2O < 0.1 ppm, O_2 < 0.1 ppm) at the temperature that gave rise to the peak circular dichroism. The film thickness was measured using a Bruker Dektak® Stylus Profilometer.

Thin film Photophysical and Morphological Characterisation: Absorption and PL spectra of the thin films were measured by a Cary 300 UV–Vis spectrometer (Agilent Technologies) and an FLS 1000 (Edinburgh Instruments), respectively.

Thin film Circular Dichroism and Circularly Polarised Photoluminescence: Steady-state (room temperature) Circular Dichroism measurements were performed using a Chirascan Plus (Applied Photophysics). For the annealed films that demonstrate strong chiroptical activity (> 2,000 mdeg), a correction factor was applied to calculate the accurate CD_{mdeg}.[3] *In situ* temperature dependent CD measurements were carried out in an N_2 environment at the B23 beamline at the Diamond Light Source using a motorised temperature controller Linkam MDS600 stage.[4,5] The heating/cooling rate was 10 °C/minute and the thin films were held for 60 seconds before spectra were acquired. Ellipticity in millidegrees can be simply calculated using $CD_{mdeg} = CD_{\Delta A} \times 32982$.

Left-handed (LH) and right-handed (RH) CP emission spectra from the ACP:oxa[7]H blend thin films were collected using a linear polarizer and zero-order quarter-wave plate (λ = 546 nm, Thorlabs WPQ05M-546) prior to the detector of an FLS 1000 spectrometer. See reference [6] for further details. The unpolarised excitation source (λ_{ex} = 385 nm unless otherwise stated) and detector were fixed at 45° from the thin film. The dissymmetry factor *g* in the CD and CP-PL spectra was calculated from;

$$g = 2\frac{I_{LH} - I_{RH}}{I_{LH} + I_{RH}}$$

Here, I_{LH} and I_{RH} refer to the LH and RH absorption/emission intensities.

Spectroscopic Ellipsometry: Mueller matrix spectroscopic ellipsometry measurements were performed using a J.A. Woollam V-Vase ellipsometer, which uses a silicon CCD with wavelength spacing of 1 nm. Data were collected in transmission and reflection using a collimated beam with diameter of 3–4 mm.

Neat oxa[7]H

Solution UV-Vis/ PL measurements

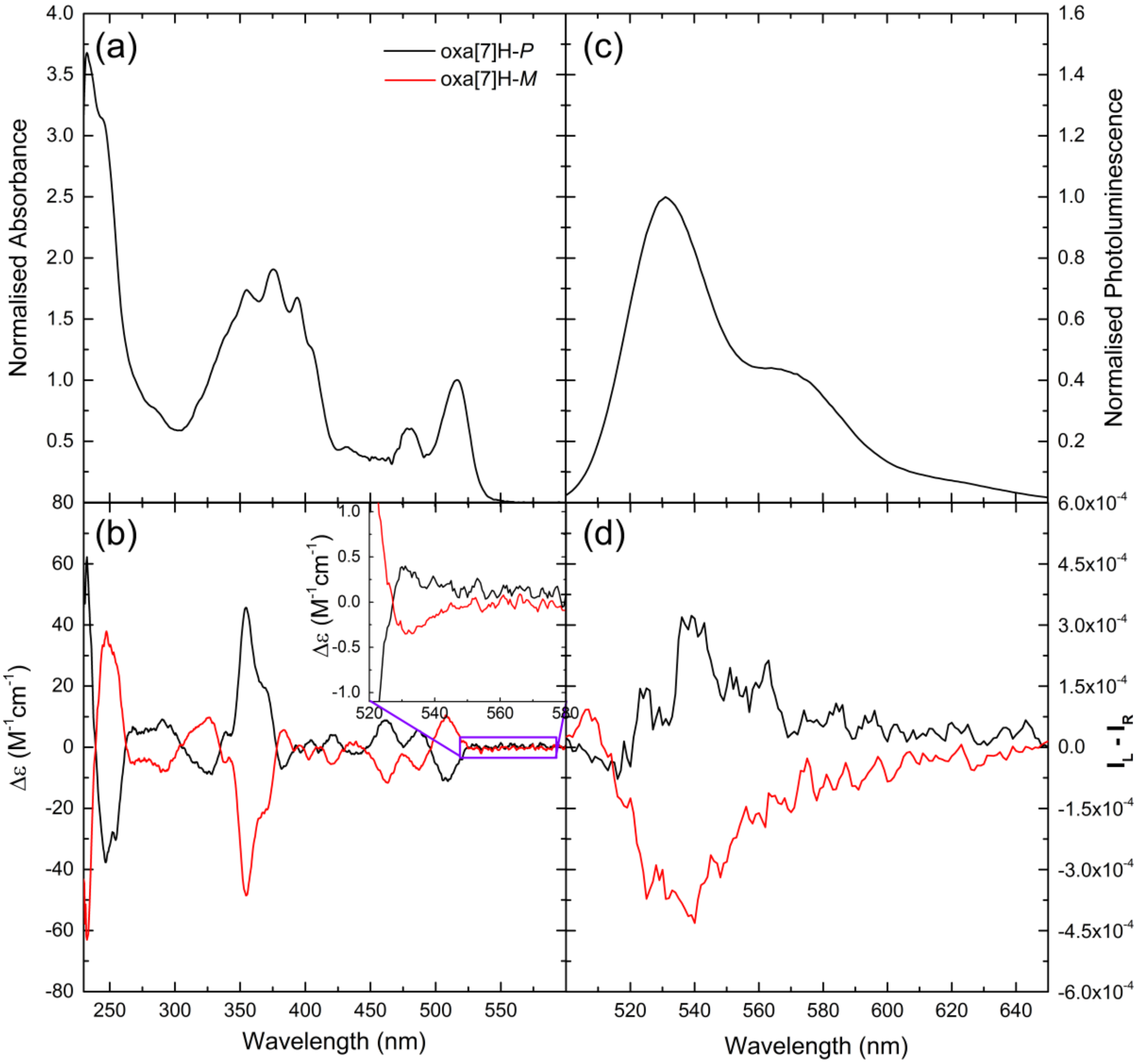

Figure S 1: (a) UV-Vis and (b) ECD spectra of oxa[7]H solutions (the inset shows a zoomed-in version of the low energy part of the ECD spectrum), (c) total fluorescence and (d) CP PL (LH – RH) of a 3 × 10^{-6} M solution in CH_2Cl_2.

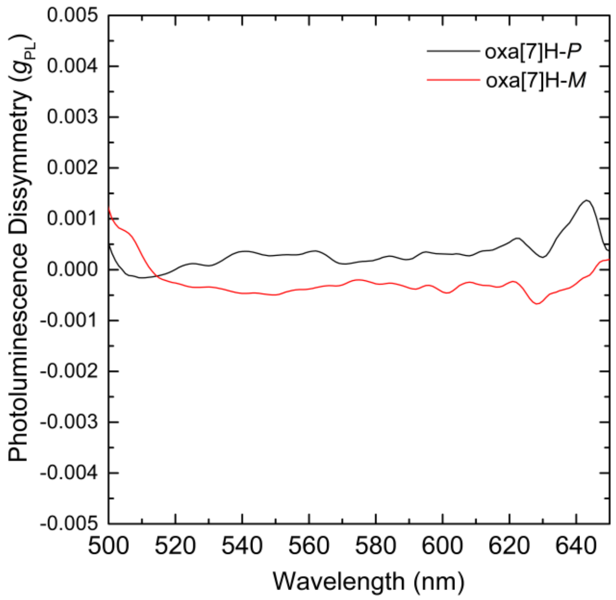

Figure S 2: Photoluminescence dissymmetry of a 3 × 10^{-6} M solution of oxa[7]H in CH_2Cl_2.

Thin film UV-Vis/ PL measurements

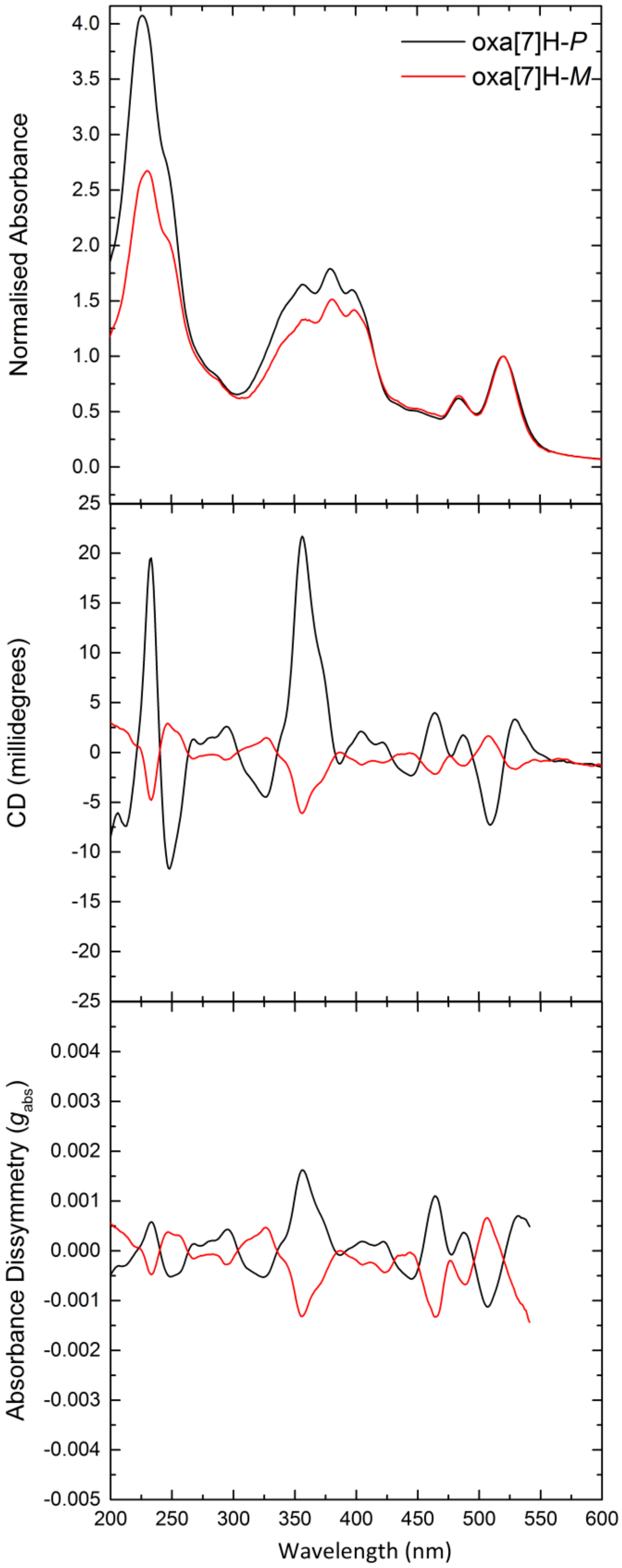

Figure S 3: UV-Vis, Circular Dichroism and the associated g_{abs} spectra of thin films (t = 90 nm) of oxa[7]H *[P]* (black) and *[M]* (red).

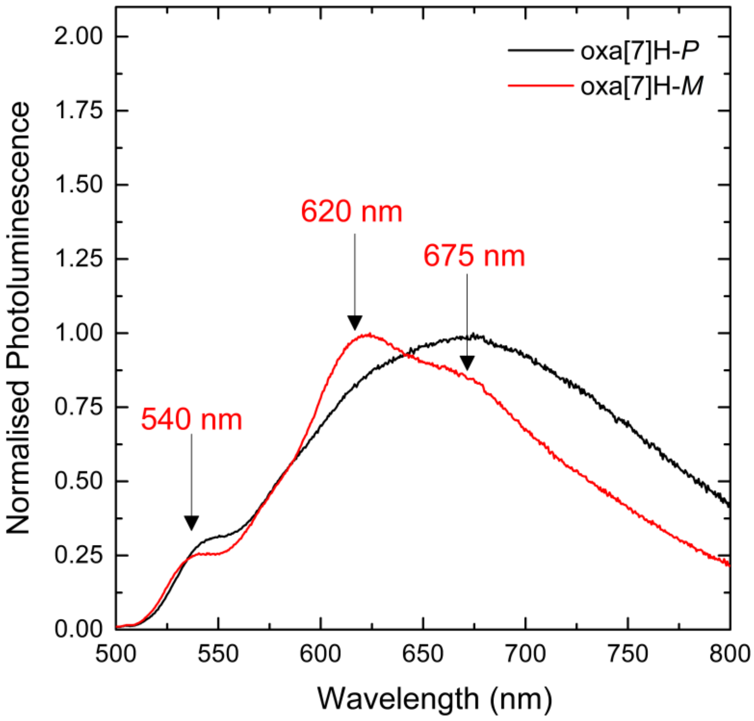

Figure S 4: Photoluminescence of thin films of oxa[7]H (t = 90 nm) of oxa[7]H *P* (black) and *M* (red) excited at λ_{ex} : 350 nm.

Materials used in this work

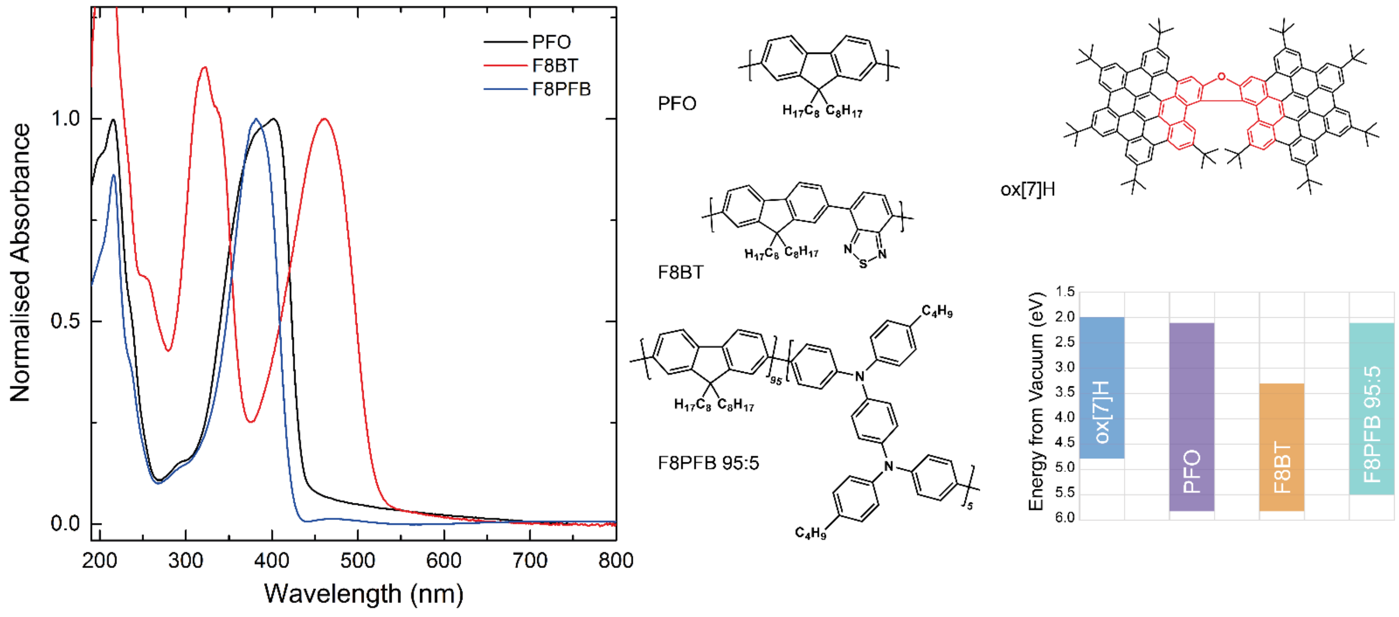

Figure S 5: Neat polymer absorption spectra, chemical structures and HOMO/LUMO levels.

PFO:oxa[7]H Blends
Photoluminescence

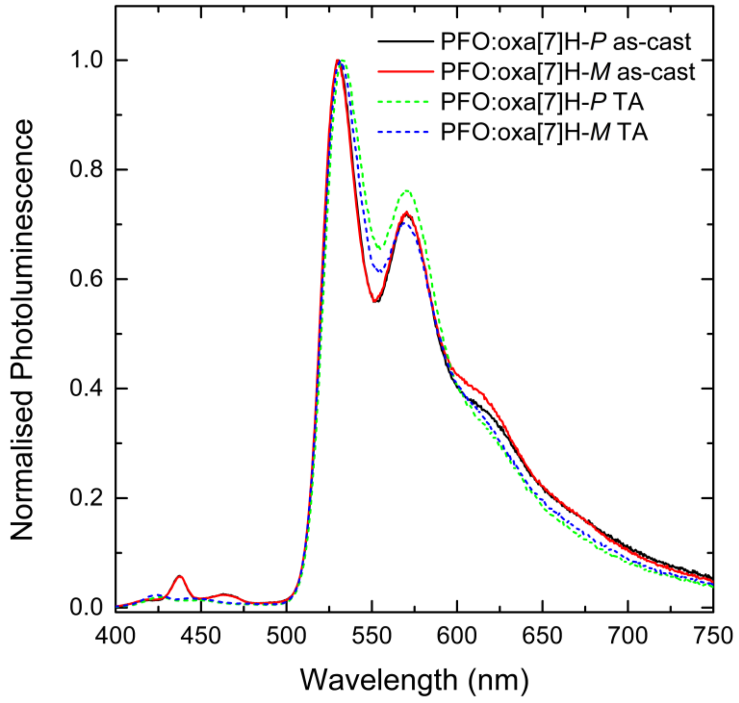

Figure S 6: Photoluminescence of as-cast (black, red) and annealed (green, blue) thin films of PFO:oxa[7]H (t = 135 nm) excited at λ_{ex} : 385 nm.

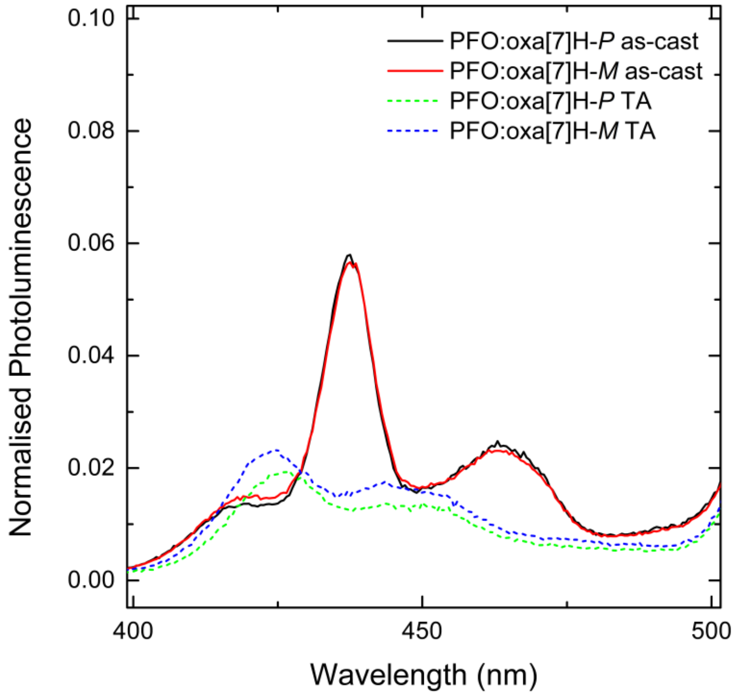

Figure S 7: Zoomed in photoluminescence of as-cast (black, red) and annealed (green, blue) thin films of PFO:oxa[7]H (t = 135 nm) excited at λ_{ex} : 385 nm.

In situ Circular Dichroism

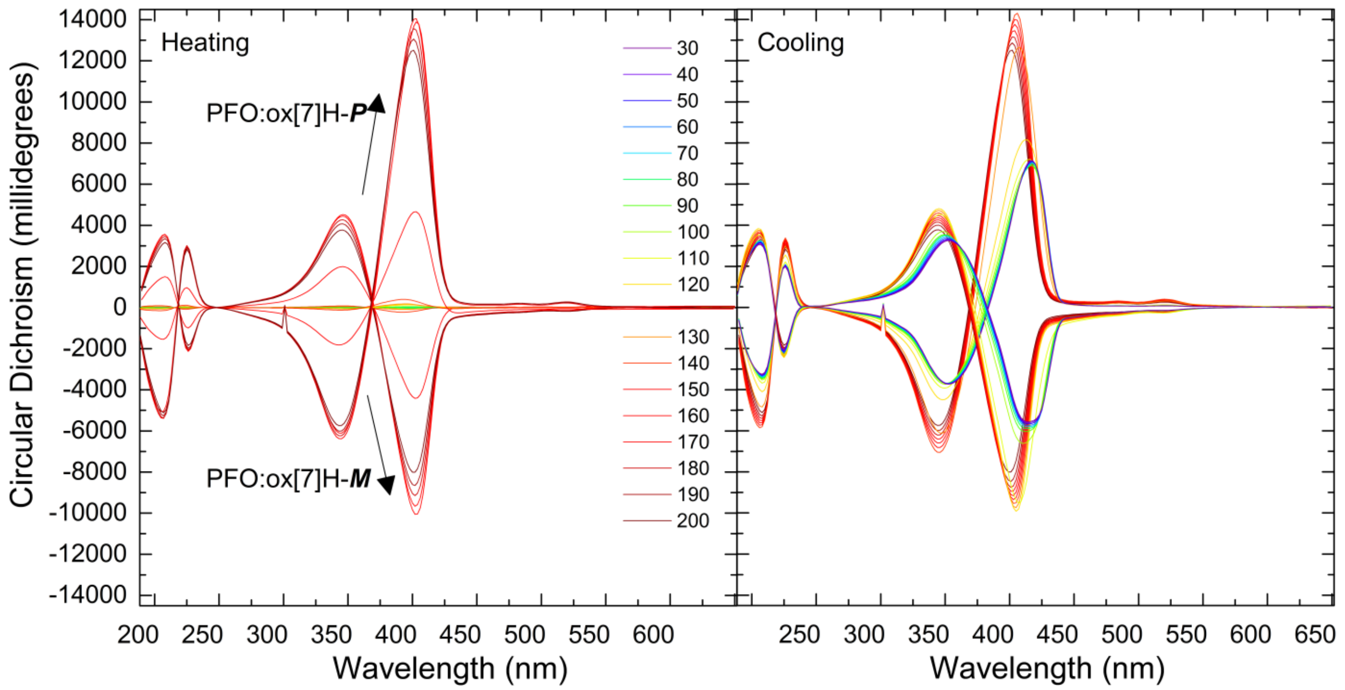

Figure S 8: *In situ* CD spectra of PFO:oxa[7]H blend thin films (t = 135 nm) recorded during heating (left) and cooling (right) from 20 to 200 °C.

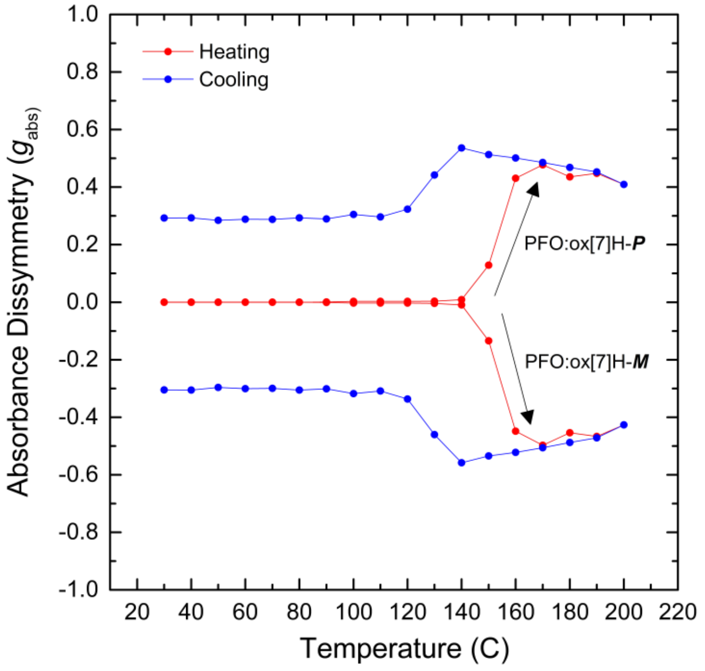

Figure S 9: g_{abs} of the low energy band in the *in situ* PFO:oxa[7]H CD spectra (Figure S8) recorded during heating and cooling from 20 to 200 °C.

Circular Dichroism

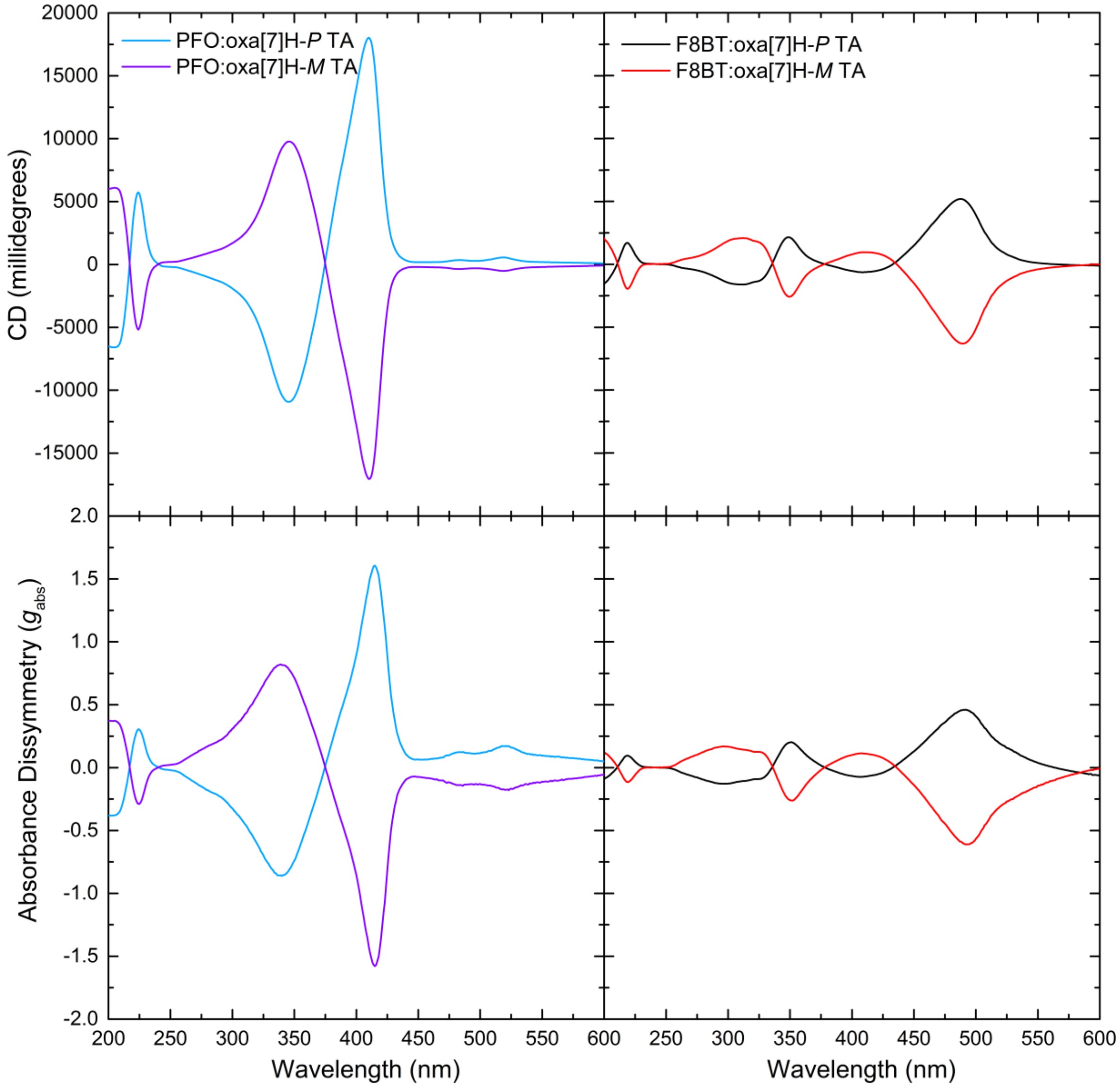

Figure S 10: CD and g_{abs} for PFO (t = 135 nm) and F8BT:oxa[7]H (t = 131 nm) thin films annealed for 10 minutes in an N_2 environment at 160 °C and 140 °C respectively.

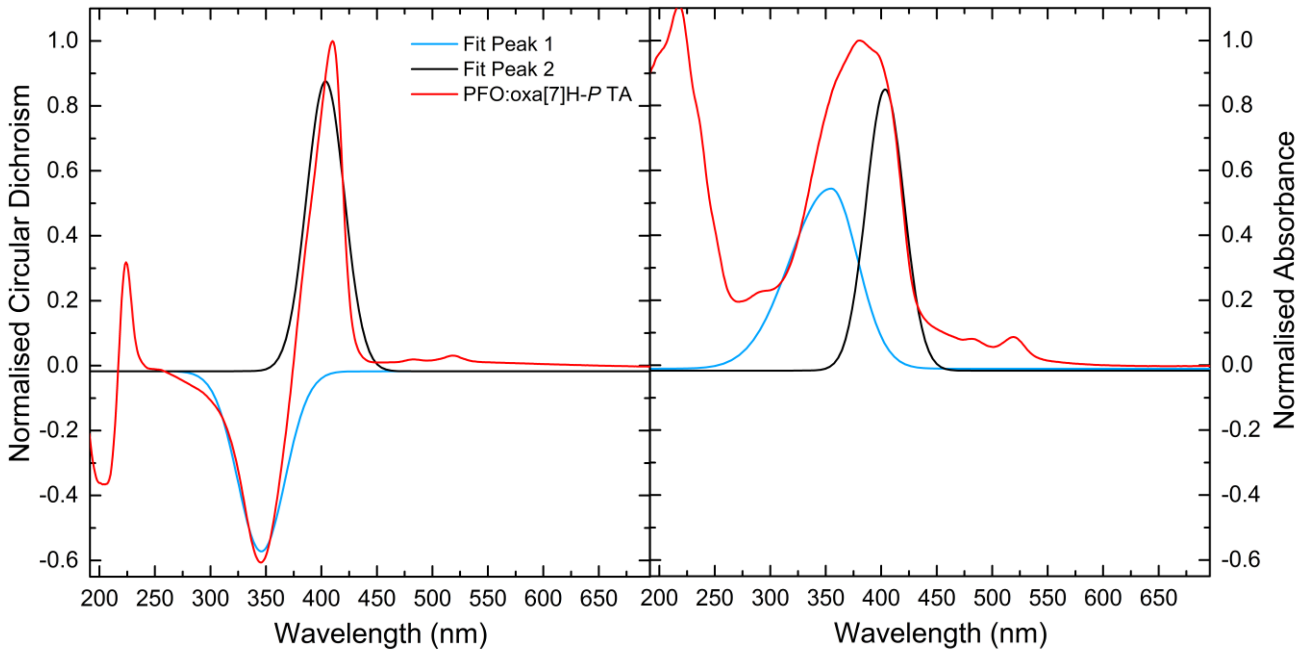

Figure S 11: Excitonic coupling in annealed PFO:oxa[7]H-*P* blend thin films (t = 135 nm) gives rise to an intense bisignate couplet centred at the absorption maximum.

The strength of the CD response (Figure S10) and extent of this exciton coupling (Figure S11) implies that the PFO chromophores may possess no local symmetry, in which case, transitions are magnetically and electrically allowed.[7] Moreover, it is well known that strong coupling between dissymmetically juxtaposed groups will itself give rise to a chiral response through the E1 transition moments alone.[8] As for the MMSE data (below), the strong circular terms recorded in transmission but not reflection, and absence of any significant linear terms in transmission spectra (Figure S12 – S13), imply that the strong chiroptical response arises due to natural optical activity, not structural chirality.[9] These results imply that PFO:oxa[7]H systems adopt a double twist cylinder blue phase comparable with our previous work with ACPCA systems.[9]

Mueller Matrix Spectroscopic Ellipsometry

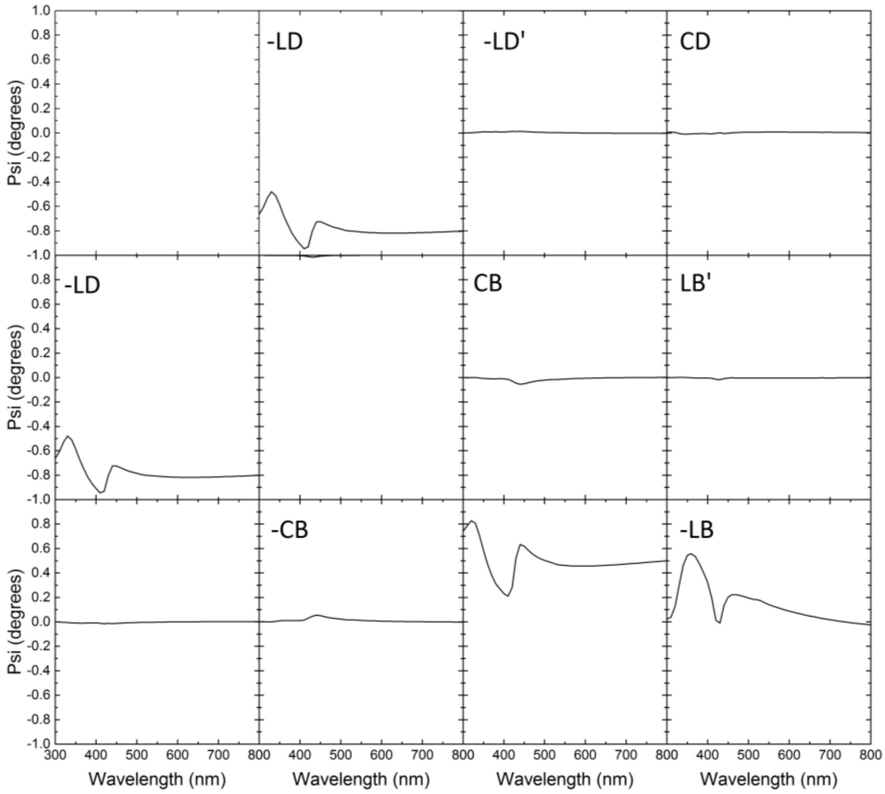

Figure S 12: Reflection MMSE spectra (recorded at 70°) of annealed PFO:oxa[7]H-*M* thin films (t = 135 nm).

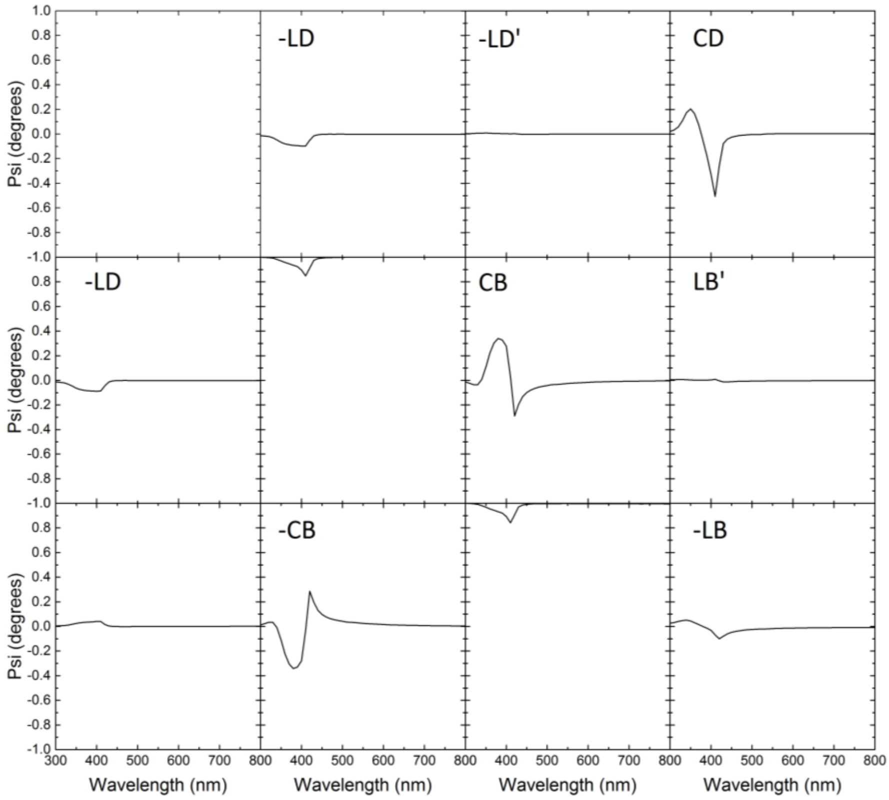

Figure S 13: Transmission MMSE spectra (recorded at 0°) of annealed PFO:oxa[7]H-*M* thin films (t = 135 nm).

PLQY measurements

Table S1: PLQY calculated following the protocol of de Mello and co-workers.[10]

	**PFO:oxa[7]H-P**		**PFO:oxa[7]H-M**	
	Donor emission	Acceptor emission	Donor emission	Acceptor emission
**as-cast**	3.0 %	49.3 %	3.0 %	49.5 %
**annealed**	3.6 %	52.1 %	3.9 %	53.2 %

CP PL measurements

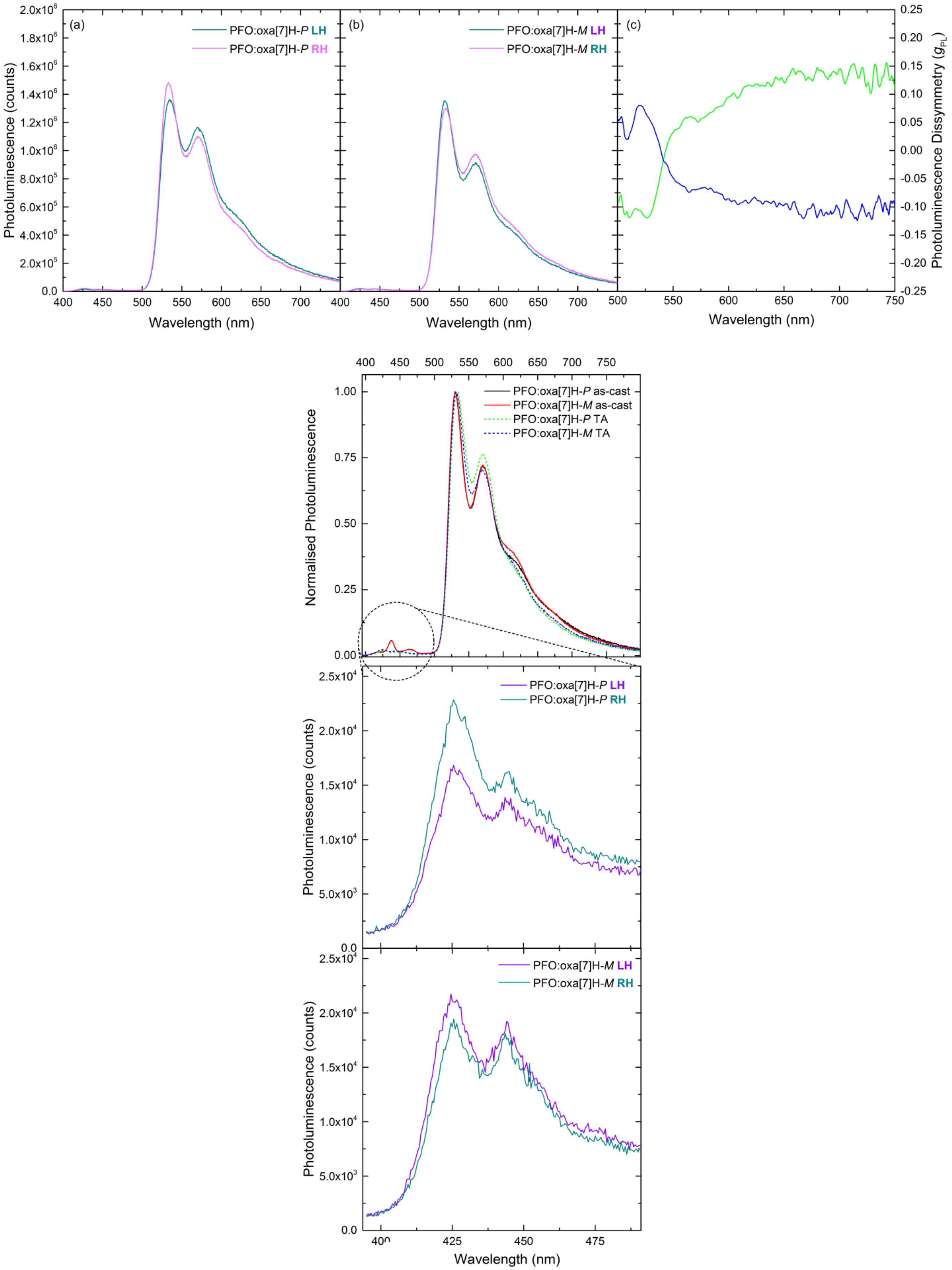

Figure S 14: CP PL spectra of PFO:oxa[7]H blends and zoomed-in section highlighting PFO emission

As illustrated in Figure S11, exciton coupling between nearby chromophores results in a split excited state, such that LH and RH emission occur from non-degenerate energy levels. A slight shift in energies can be seen in the LH and RH CPL of oxa[7]H. As can be seen in the inset figure (expanded from Figure S24, the intensity of RH CPL is slightly > LH CPL at λ < 535 nm (marked with a blue asterisk), and LH is > RH at λ > 535 nm, resulting in a bisignate CPL response.

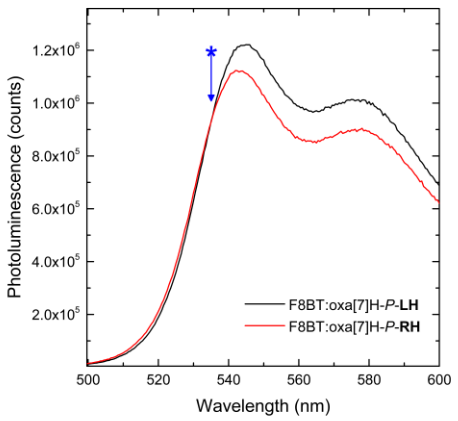

In addition to this the oxa[7]H **absorbs** CPL between 500 and 540 nm (Figure 3(c), Figure S1); and, as such, competition between the dissymmetric PFO:oxa[7]H blend **absorption** and oxa[7]H **emission** (~ 534 nm), can further give rise to an apparent bisignate response in the CPL/g_{PL} spectra. A full quantitative analysis of the light being absorbed/emitted from the system is beyond the scope of the current work, but is something that we will be pursuing in the future.

It is interesting to note that the weak emission of PFO (< 8% of integrated PL) in the as-cast and annealed PFO:oxa[7]H blends can be observed in the high energy (400–450 nm) region of the PL spectra. Annealing causes the PL peaks to blue-shift, indicating the PFO adopts a polycrystalline phase[11], and the emission is strongly dissymmetric, with the same handedness as the emission of the oxa[7]H ($|g_{PL}| \approx 0.25$, Figure S14).

In the case of the annealed PFO:oxa[7]H films, the broadened absorption band and bisignate CD couplet centred close to the PFO absorption maximum (375 nm) is the result of split excited states, with the strong, bisignate CD response (Figure S11 and accompanying discussion) resulting from the non-orthogonal E1 and M1 transition dipole moments on adjacent PFO chromophores.[8,12]

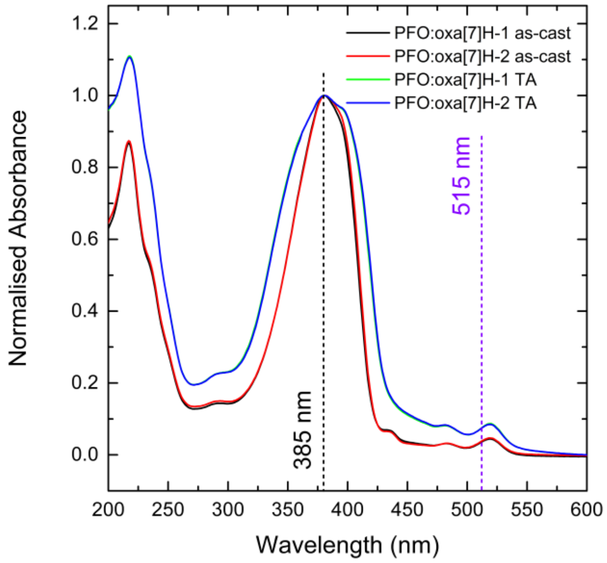

Figure S 15: Absorption spectrum of PFO:oxa[7]H blend thin films (t = 135 nm) showing PFO (λ_{abs} : 385 nm) and oxa[7]H (λ_{abs} : 515 nm) absorption bands

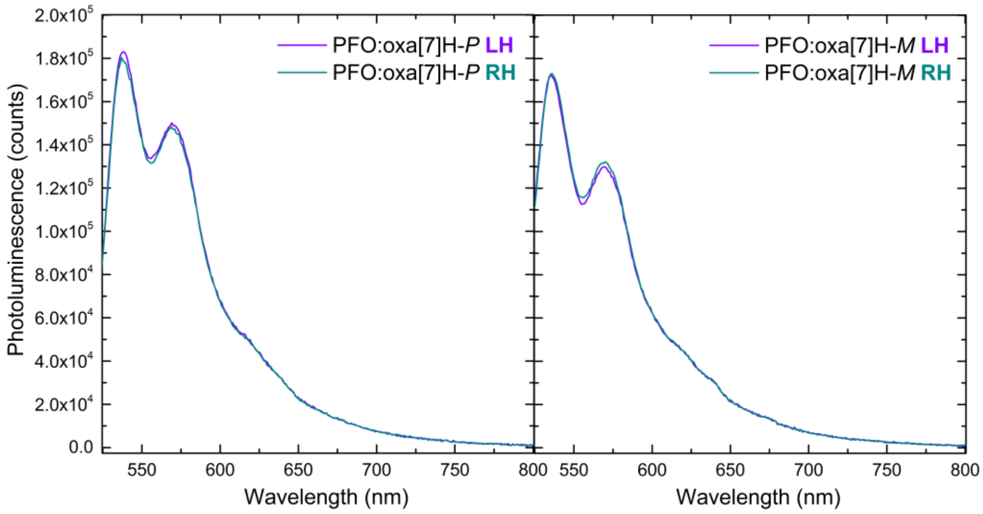

Figure S 16: CP PL spectra for PFO:oxa[7]H-***P*** (left) and oxa[7]H-***M*** (right) systems acquired at λ_{ex} : 515 nm

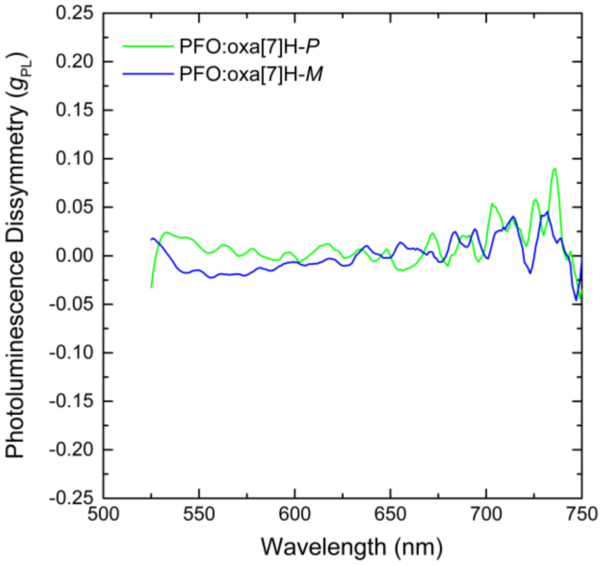

Figure S 17: g_{PL} for PFO:oxa[7]H-***P*** (green) and oxa[7]H-***M*** (blue) acquired at λ_{ex} : 515 nm.

Atomic Force Microscopy

Figure S 18: Atomic Force Microscopy images of annealed PFO and PFO:oxa[7]H thin films.

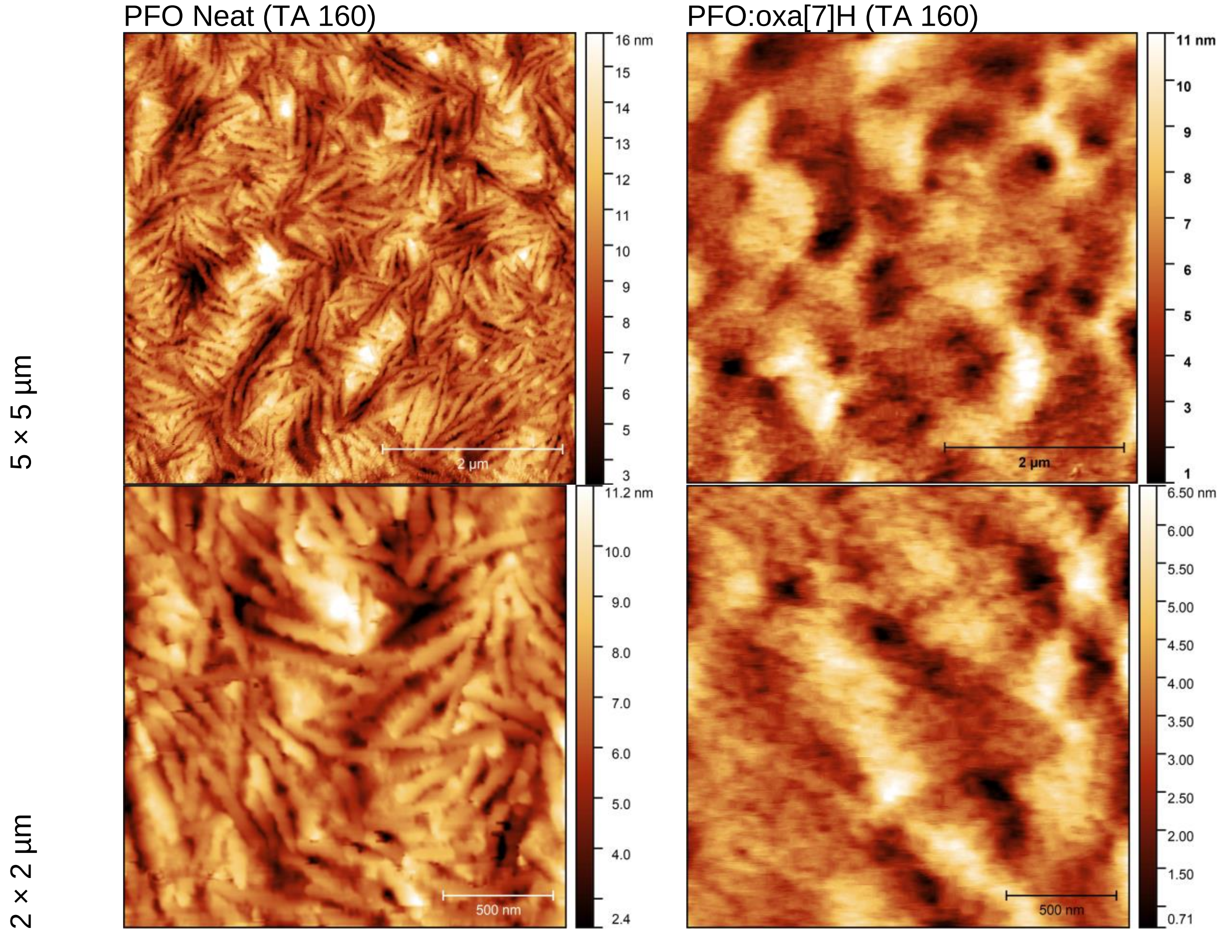

Topographic AFM images of neat and blend PFO

AFM images of annealed neat PFO and PFO:oxa[7]H thin films indicate dramatically different topographies. Thin films of neat PFO comprise of distinct fibrils, 2 – 3 nm tall, 100 nm wide and 100s of nm long. These fibrils show no preferred orientation. On the other hand, no such structures are seen in the PFO:oxa[7]H blend films; which, comparable to our previous study using aza[6]H, are more smooth than their neat polymer counterparts (neat PFO, root-mean-square roughness, r_q = 0.6 nm and PFO:oxa[7]H, r_q = 0.2 nm).

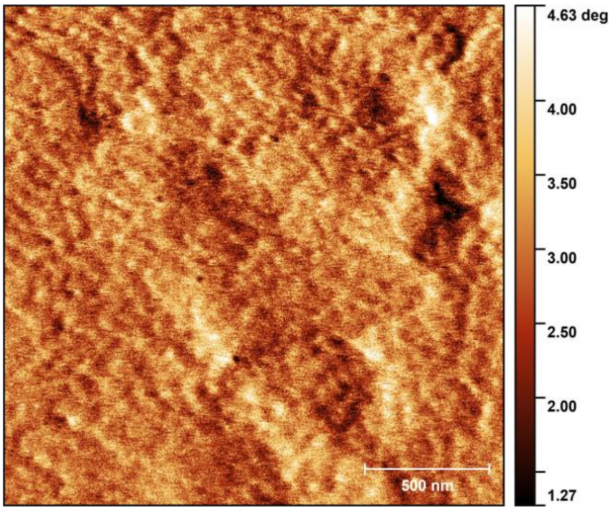

Further analysis of the phase images of the annealed PFO:oxa[7]H system reveals that the surface comprises of distinct circular voids, separated by ~ 300 nm, which are reminiscent of the dislocations in the double twist cylinder blue phase thin films we previously observed in aza[6]H ACPCA blends.[9]

Excited state dynamics

To better understand the specific type of energy transfer that has taken place in the blend systems we examined the fluorescence decay kinetics using Time Correlated Single Photon Counting (TCSPC). As can be seen in the photoluminescence spectra, the fluorescence intensity of PFO decreases dramatically in the blend system. We also observe a decrease in the fluorescence lifetime of PFO (λ = 435 nm) in the PFO:oxa[7]H blends compared to their neat (τ ~ 160ps) counterparts, to around 1/5th of the original value (τ ~ 30ps). Unfortunately, these time scales are near the limits of our detector, and therefore accurate calculation of the energy transfer efficiency is not possible.[13] It is interesting to note that the acceptor (oxa[7]H) in the PFO:oxa[7]H blend system has a considerably longer lifetime than the donor, closer to 1 ns.

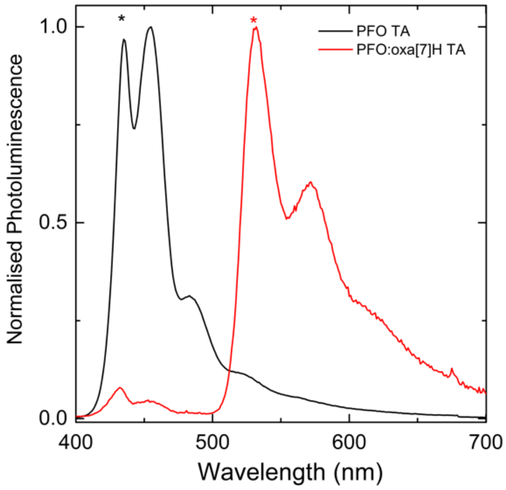

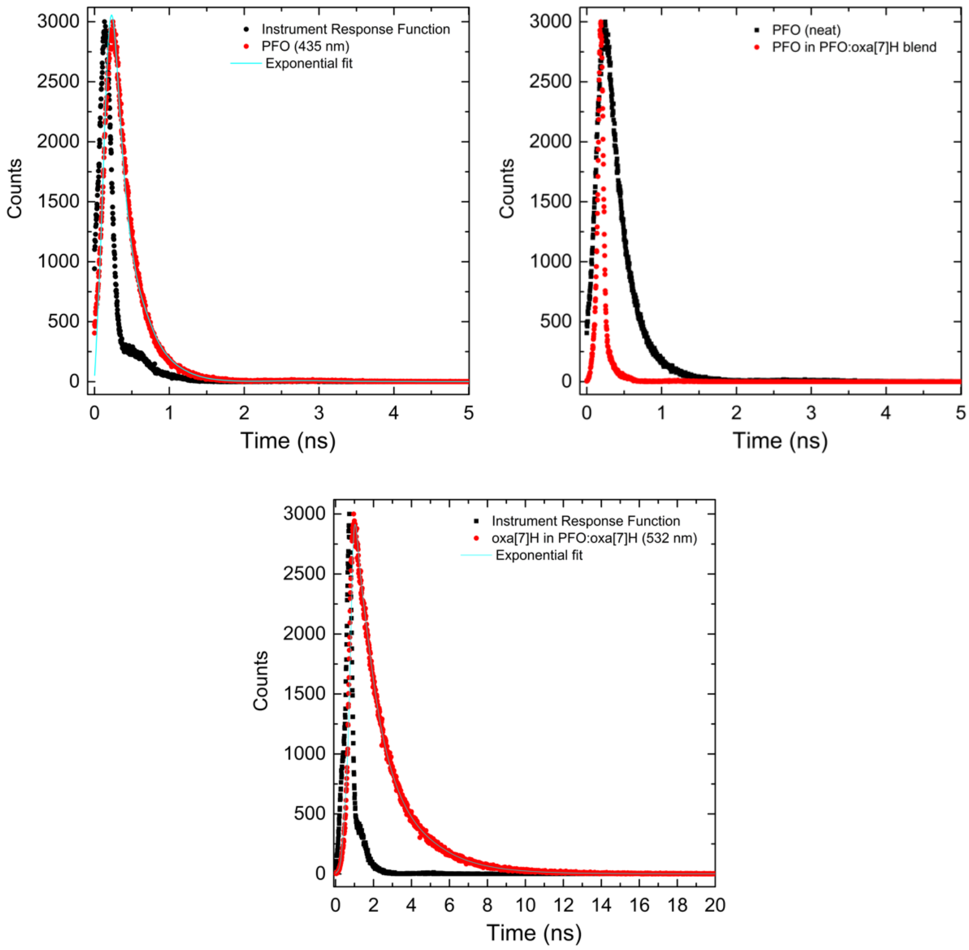

Figure S 19: Time correlated fluorescence decay of PFO in neat and PFO:oxa[7]H blend systems, as well as that of the acceptor (oxa[7]H).

F8BT:oxa[7]H

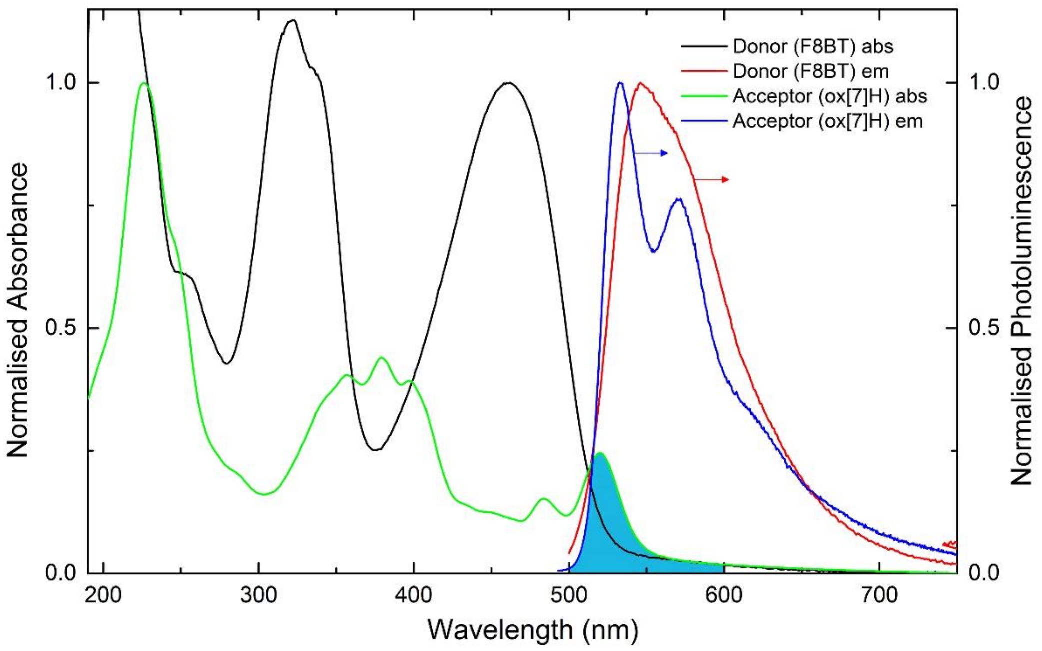

Figure S 20: Absorption and photoluminescence spectra of the donor (F8BT) and acceptor (oxa[7]H) systems. The overlap between the donor emission and acceptor absorption is highlighted.

In situ Circular Dichroism

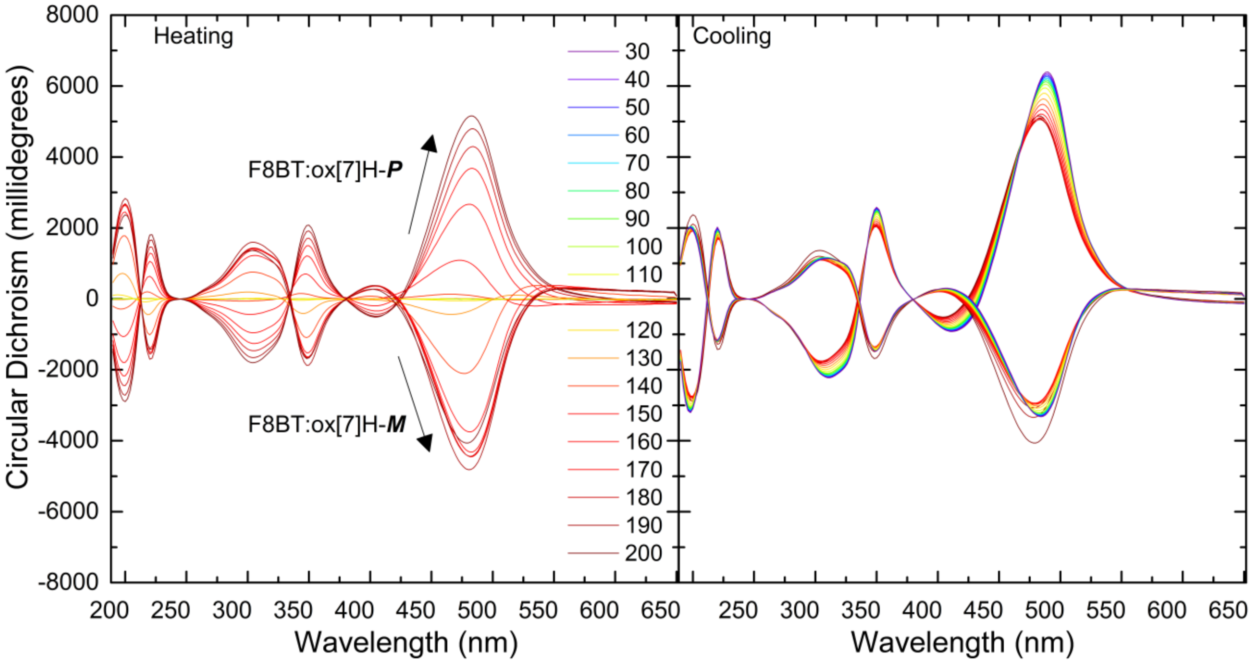

Figure S 21: *In situ* CD spectra of F8BT:oxa[7]H blend thin films (t = 131 nm) recorded during heating (left) and cooling (right) from 20 to 200 °C.

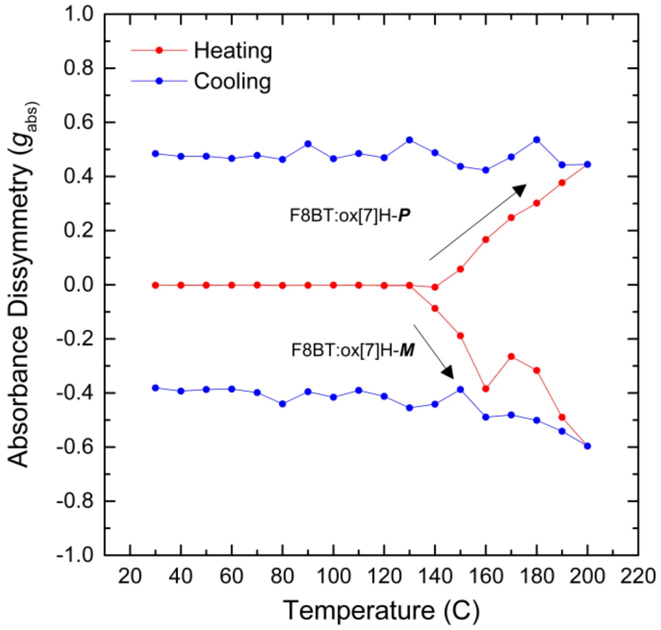

Figure S 22: g_{abs} spectra of the low energy CD band of the F8BT:oxa[7]H blend thin films (Figure S19) recorded during heating and cooling from 20 to 200 °C.

Dissymmetric absorption/photoluminescence

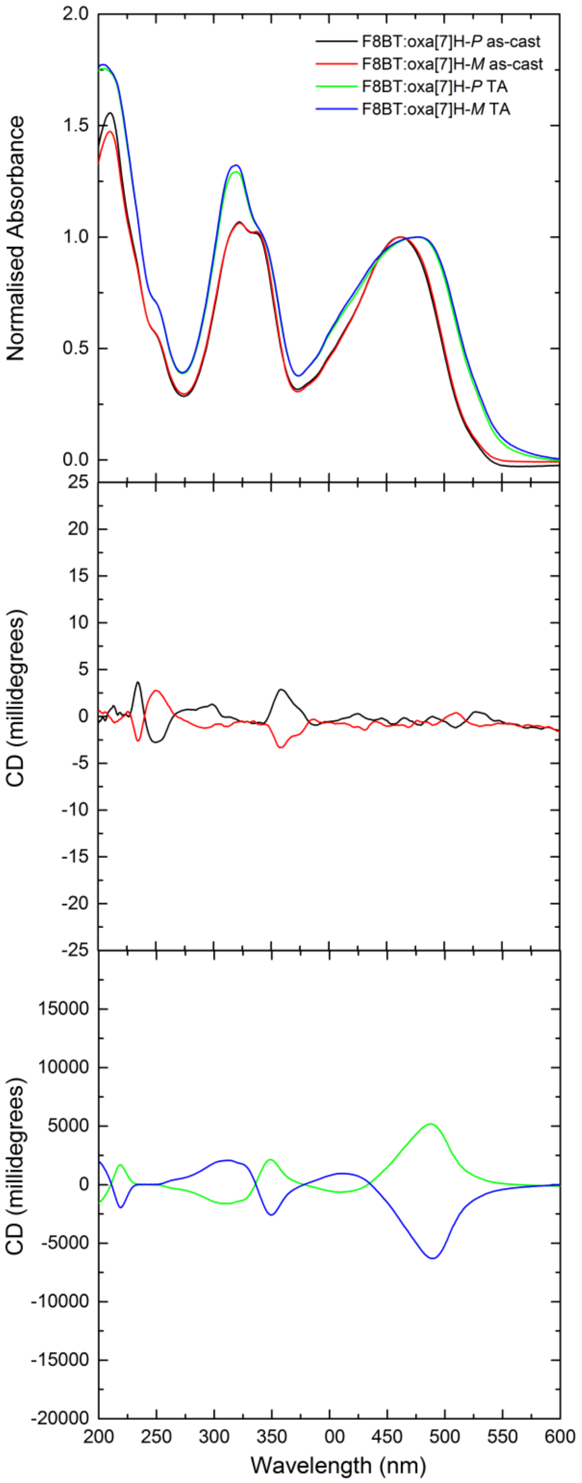

Figure S 23: UV-Vis and Circular Dichroism spectra of as-cast (black, red) and annealed (green, blue) F8BT:oxa[7]H thin films (t = 131 nm).

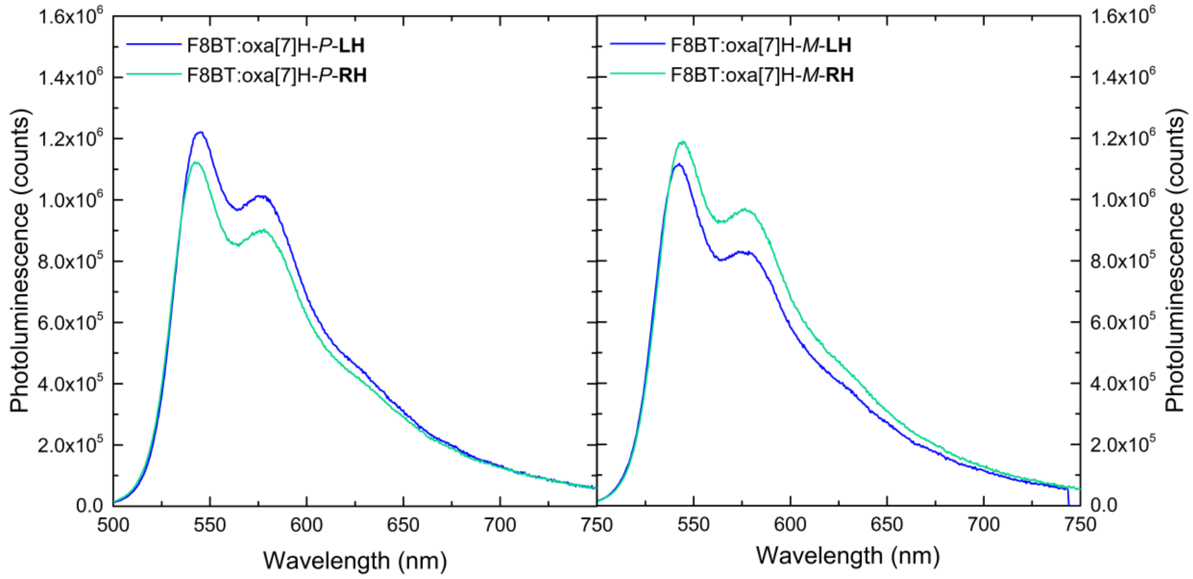

Figure S 24: CP PL spectra for F8BT:oxa[7]H-***P*** (left) and F8BT:oxa[7]H-***M*** (right) systems acquired at λ_{ex} = 475 nm

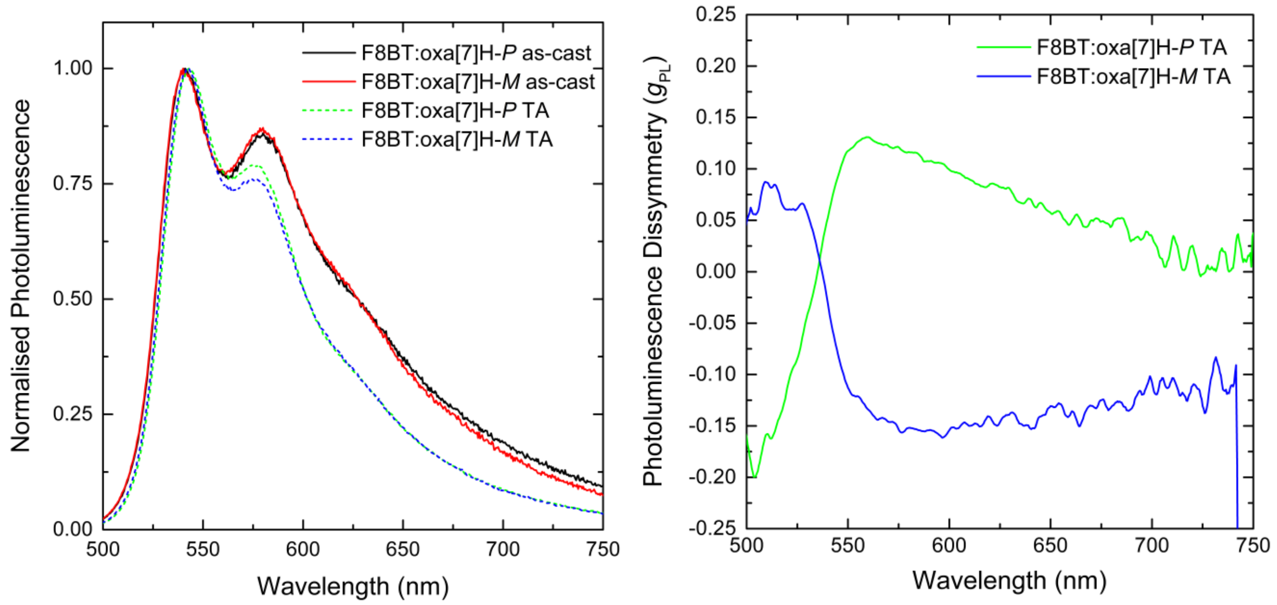

Figure S 25: PL and g_{PL} for F8BT:oxa[7]H-***P*** (green) and F8BT:oxa[7]H-***M*** (blue) acquired at λ_{ex} = 515 nm

Discussion of F8BT results

In the case of F8BT:oxa[7]H (Figure S18), annealing causes an overall broadening of the absorption bands, as well as a clear increase in the oscillator strength of the high energy absorption bands and red-shift of the low energy absorption and (λ_{abs} as-cast: 461 nm, annealed: 482 nm). *In situ* CD spectra (Figure S19 – S20) reveal a considerably different phase behaviour to our previous work with aza[6]H, with $T_{CD\ Max} \simeq 200$ °C and no significant loss of the CD response on cooling to room temperature. As the low energy oxa[7]H overlap with the low energy F8BT band, it is hard to distinguish oxa[7]H from F8BT in the CD spectra (Figure S21), but the influence of oxa[7]H is evident in the strong, mirror image CD bands (CD ≈ 6,000 mdeg) of otherwise achiral F8BT. As with the PFO:oxa[7]H blends, the results imply the formation of a similar double twist cylinder phase to our previous work with ACPCA systems.[9] In this case, the low energy (λ_{abs} = 487 nm) CD band is strong, with weak evidence of exciton coupling, whereas the higher energy transitions are comprised of bisignate couplets centred at the transition of the isolated chromophores (λ_{abs} = 335 and 210 nm).

Comparing the UV-Vis spectra of the annealed F8BT:oxa[7]H films with their as-cast counterparts (Figure S21), the increase in oscillator strength of the high energy transitions (λ_{abs}: 335 and 210 nm) suggests that in the chiral phase the polymer chains adopt a more in-plane orientation, which results in a redistribution of the oscillator strengths of absorbing dipoles along the F8BT backbone.[14,15] It is interesting to note that the low energy, charge-transfer like absorption band displays weak evidence of exciton coupling, but the high energy absorption bands, which correspond to more delocalised transitions, exhibit bisignate CD responses centred at the absorption maxima (λ_{abs}: 335 and 210 nm). The red-shifted peaks and slightly more intense $|g_{PL}|$ for the F8BT:oxa[7]H compared to PFO:oxa[7]H thin films may be the result of the considerable overlap of the oxa[7]H PL with that of the donor F8BT (Figure 22 – 23).

F8:PFB:oxa[7]H

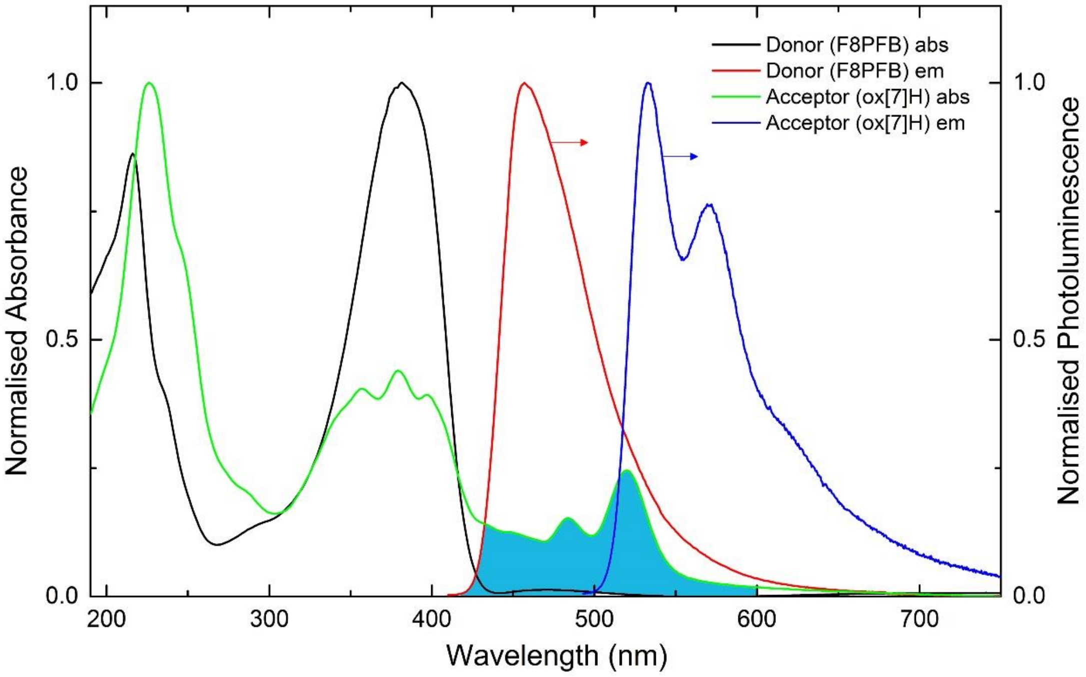

Figure S 26: Absorption and photoluminescence spectra of the donor (F8PFB) and acceptor (oxa[7]H) systems. The overlap between the donor emission and acceptor absorption is highlighted.

UVVis/PL

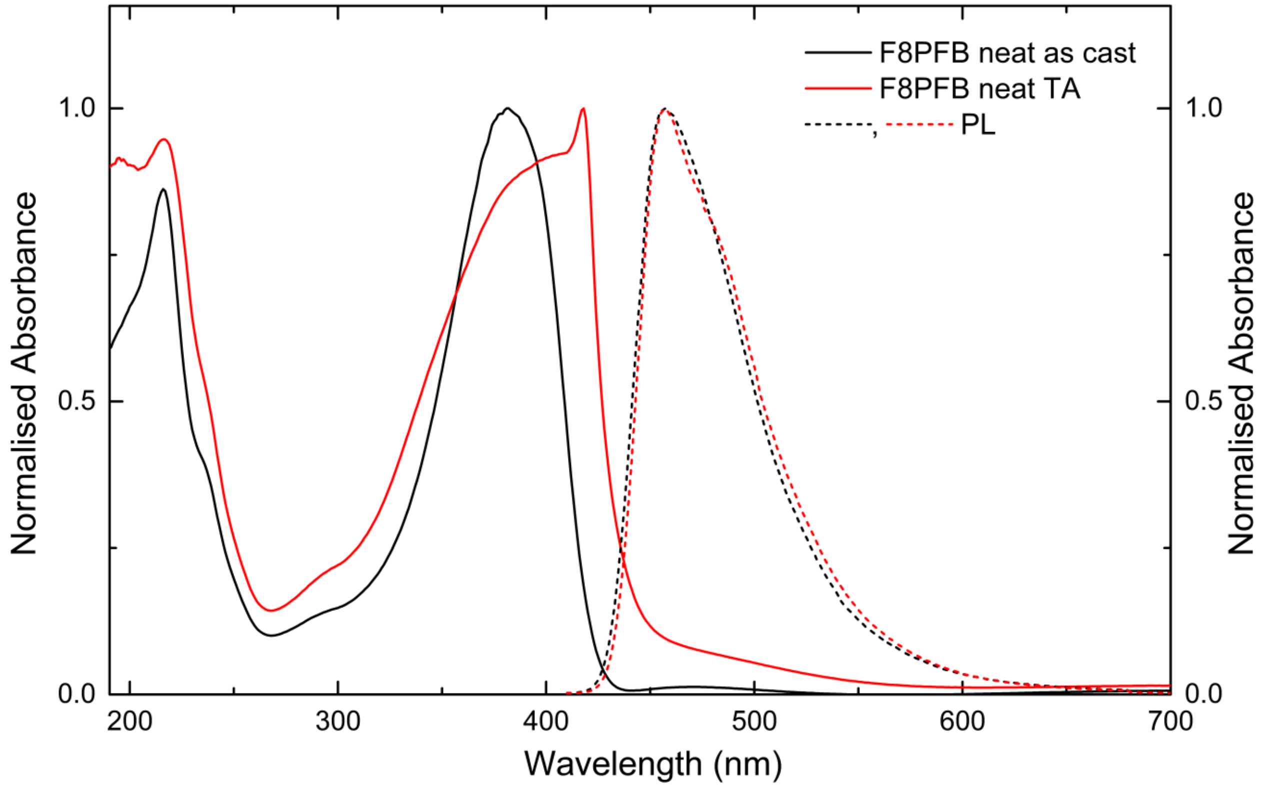

Figure S 27: UV-Vis and PL of neat (as-cast and annealed) films of F8PFB (95:5).

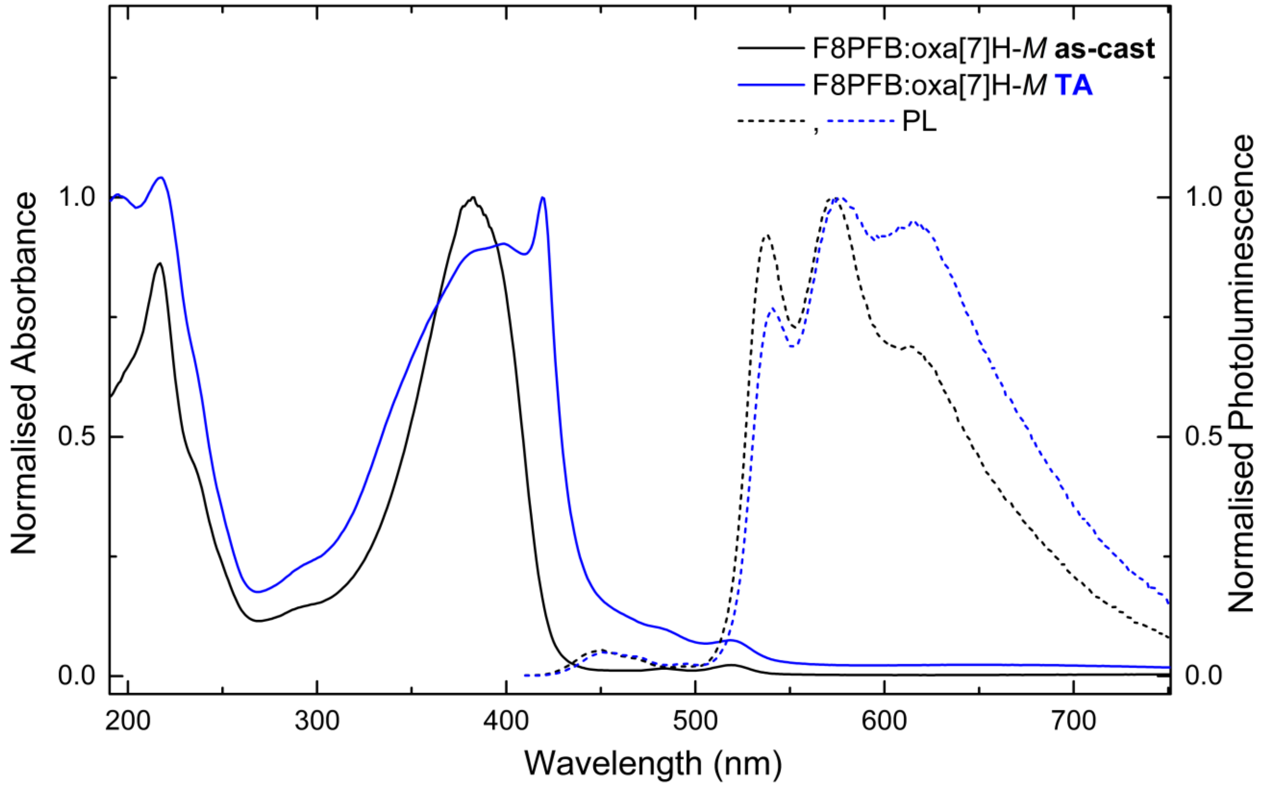

Figure S 28: UV-Vis and PL of F8PFB:oxa[7]H-***M*** (as-cast and annealed) thin films (t = 137 nm). The annealing temperature was 160 °C.

PLQY measurements

Table S2: PLQY calculated following the protocol of de Mello and co-workers.[10]

	**F8PFB:oxa[7]H-M**	
	Donor emission	Acceptor emission
**as-cast**	2.9 %	25.5%
**annealed**	2.6 %	30.0%

Dissymmetric absorption/photoluminescence

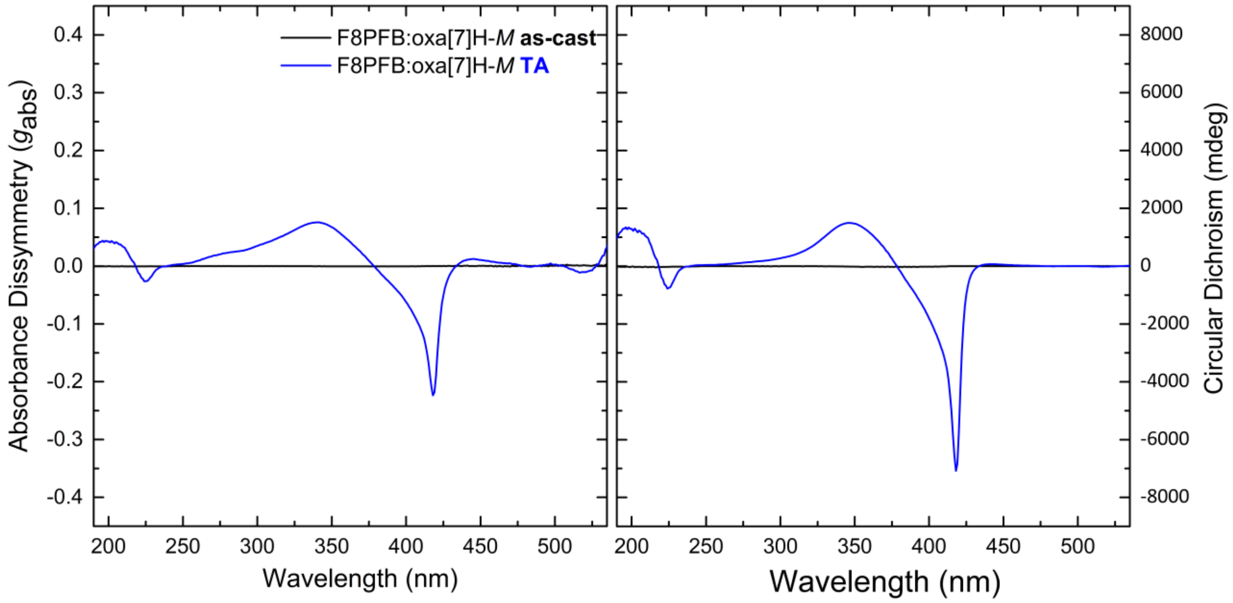

Figure S 29: CD and associated g_{abs} spectra for F8PFB:oxa[7]H-***M*** (as-cast and annealed, TA 160 °C) films (t = 137 nm).

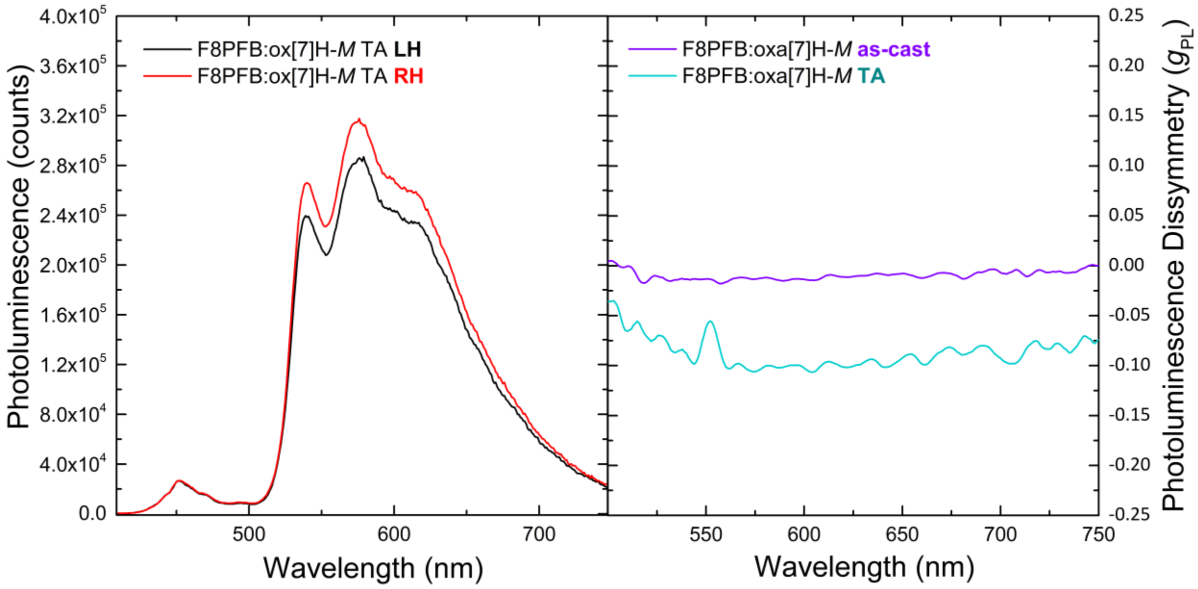

Figure S 30: CP PL and associated g-PL spectra for F8PFB:oxa[7]H (annealed at 160 °C).

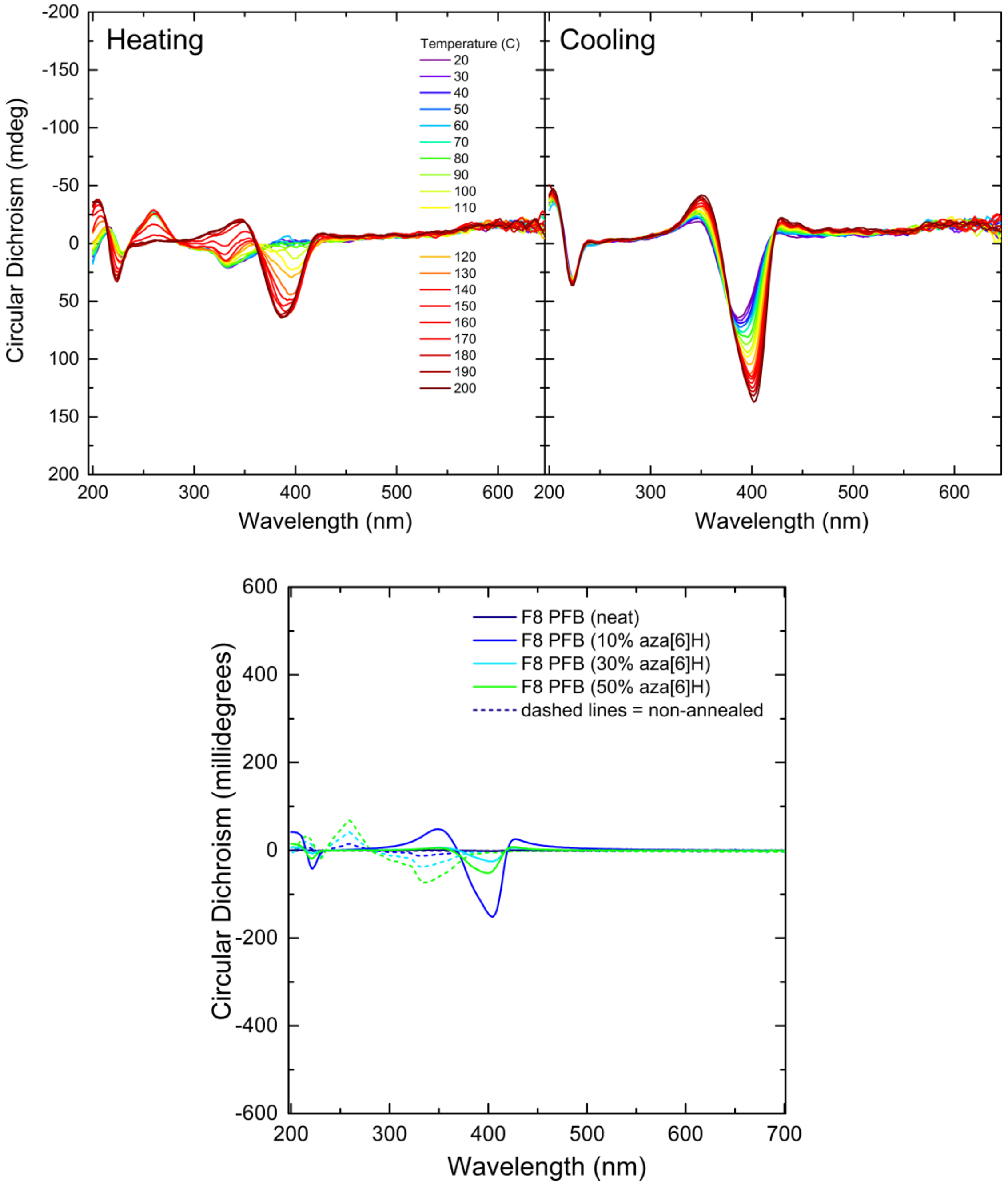

Figure S 31: (top) *In situ* CD acquired during heating and cooling of a 10 wt% F8PFB:aza[6]H (*M*) blend. (bottom) CD spectra for as-cast and annealed (160 °C) 0, 10, 30 and 50 wt% F8PFB:aza[6]H (*M*) blends.

Mueller Matrix Spectroscopic Ellipsometry Measurements

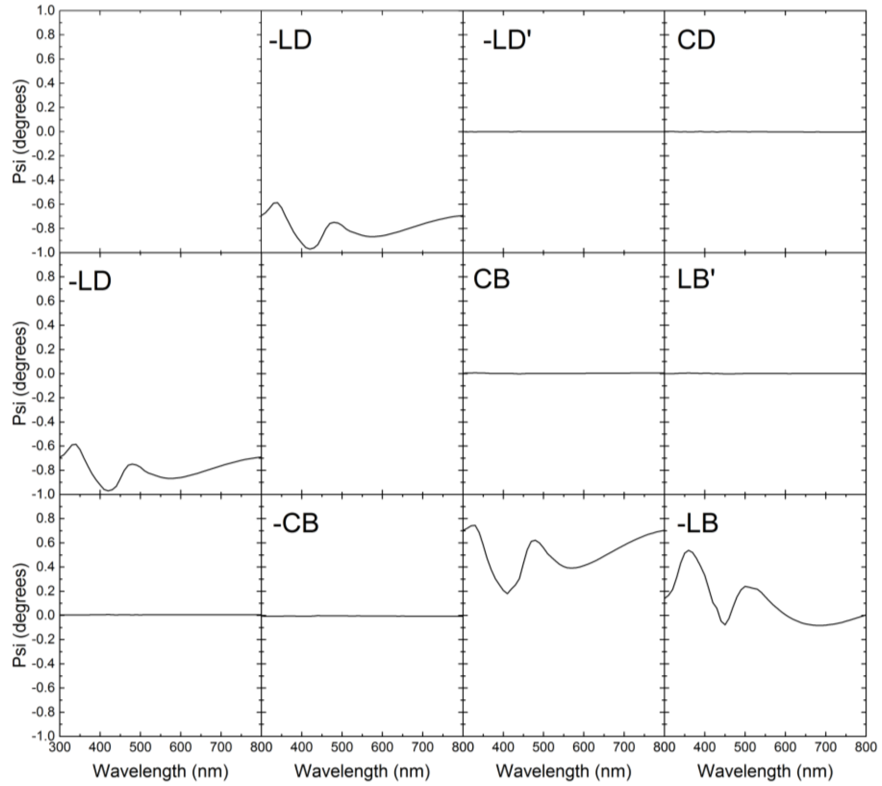

Figure S 32: Reflection MMSE spectra (recorded at 70°) of annealed F8PFB:ox[7]H-***M*** films (t = 137 nm).

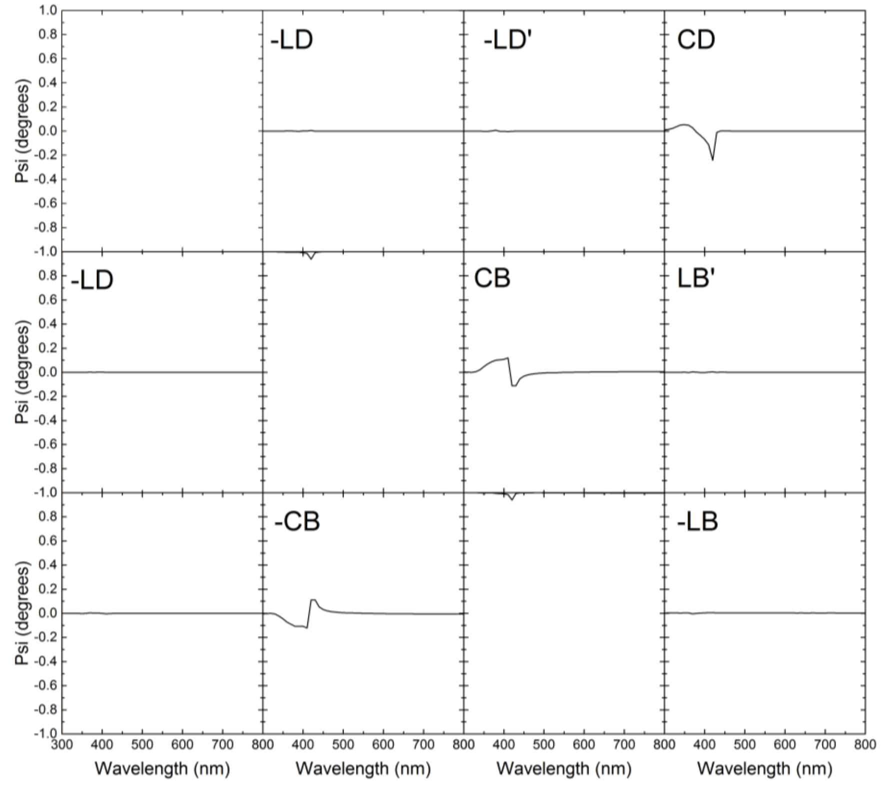

Figure S 33: Transmission MMSE spectra (recorded at 0°) of F8PFB:ox[7]H-***M*** films (t = 137 nm).

Discussion of F8PFB results

Motivated by the overlap between F8:PFB PL and oxa[7]H absorption (Figure S26), we wondered whether the larger size of the oxa[7]H may permit the formation of a chiral phase in F8:PFB. Annealing the neat polymer films results in a sharp increase of the 0 – 0 (418 nm) transition, but minimal changes to the photoluminescence spectra (Figure S27). The sharp, intense feature in the CD spectrum corresponds to the $S_1 – S_0$ 0 – 0 vibronic peak of F8PFB. The observation of this chiral phase in F8PFB is particularly interesting as it is not possible to achieve the same phase using the chiral small molecule additive aza[6]H, irrespective of annealing temperature or time (Figure S29); which we had presumed was due to the bulky butyl-substituted phenylenediamine units. MMSE measurements (Figure S31 and S32) indicate the formation of a similar double twist cylinder blue phase to that observed for the PFO:oxa[7H] thin films (Figure S12 – S13).[9]

As with the PFO and F8BT:oxa[7]H blend systems, the PL of the F8:PFB-oxa[7]H blend films is dominated by the oxa[7]H (ϕ_F ~ 30%, Figure S28, Table S2) with only a small contribution from the polymer (ϕ_F ~ 2.6%). The PLQY measurements (Table S2) indicate weaker FRET than for the PFO blends (Table S1).

In the case of F8PFB, the weaker g_{PL} and ϕ_F , absence of a bisignate CPL signal (Figure 5) and different relative intensities of the vibronic PL bands to the other ACP:oxa[7]H blends suggests aggregation of the oxa[7]H molecules.

References

[1] D. Reger, P. Haines, F. W. Heinemann, D. M. Guldi, N. Jux, *Angew. Chemie Int. Ed.* **2018**, *57*, 5938–5942.

[2] F. Zinna, U. Giovanella, L. Di Bari, *Adv. Mater.* **2015**, *27*, 1791–1795.

[3] L. Velluz, M. Le Grand, M. Grosjean, *Optical Circular Dichroism: Principles, Measurements, and Applications*, Verlag Chemie, **1965**.

[4] F. Zinna, C. Resta, M. Górecki, G. Pescitelli, L. Di Bari, T. Jávorfi, R. Hussain, G. Siligardi, *Macromolecules* **2017**, *50*, 2054–2060.

[5] G. Albano, M. Górecki, G. Pescitelli, L. Di Bari, T. Jávorfi, R. Hussain, G. Siligardi, *New J. Chem.* **2019**, *43*, 14584–14593.

[6] L. Wan, J. Wade, F. Salerno, O. Arteaga, B. Laidlaw, X. Wang, T. Penfold, M. J. Fuchter, A. J. Campbell, *ACS Nano* **2019**, *13*, 8099–8105.

[7] H.-G. Kuball, in *Encycl. Anal. Sci.* (Ed.: A. Townsend), Elsevier, **2005**, pp. 60–79.

[8] N. Berova, N. Harada, K. Nakanishi, in *Encycl. Spectrosc. Spectrom.*, Elsevier, **2017**, pp. 539–557.

[9] J. Wade, J. Hilfiker, J. R. Brandt, L. Liirò-Peluso, L. Wan, X. Shi, F. Salerno, S. Ryan, S. Schöche, O. Arteaga, T. Jávorfi, G. Siligardi, C. Wang, D. B. Amabilino, P. Beton, A. Campbell, M. Fuchter, **2020**, DOI 10.26434/chemrxiv.12277889.v1.

[10] J. C. de Mello, H. F. Wittmann, R. H. Friend, *Adv. Mater.* **1997**, *9*, 230–232.

[11] A. Perevedentsev, N. Chander, J.-S. Kim, D. D. C. Bradley, *J. Polym. Sci. Part B Polym. Phys.* **2016**, *54*, 1995–2006.

[12] N. Berova, L. Di Bari, G. Pescitelli, *Chem. Soc. Rev.* **2007**, *36*, 914–931.

[13] S. Sardar, P. Kar, H. Remita, B. Liu, P. Lemmens, S. Kumar Pal, S. Ghosh, *Sci. Rep.* **2015**, *5*, 17313.

[14] K. G. Jespersen, W. J. D. Beenken, Y. Zaushitsyn, A. Yartsev, M. Andersson, T. Pullerits, V. Sundström, *J. Chem. Phys.* **2004**, *121*, 12613–12617.

[15] J. M. Winfield, C. L. Donley, J.-S. Kim, *J. Appl. Phys.* **2007**, *102*, 063505.
